# Transmission Dynamics of Rift Valley Fever Virus: Effects of Live and Killed Vaccines on Epizootic Outbreaks and Enzootic Maintenance

**DOI:** 10.3389/fmicb.2015.01568

**Published:** 2016-02-01

**Authors:** Farida Chamchod, Chris Cosner, R. Stephen Cantrell, John C. Beier, Shigui Ruan

**Affiliations:** ^1^Department of Mathematics, Faculty of Science, Mahidol UniversityBangkok, Thailand; ^2^Department of Mathematics, University of MiamiCoral Gables, FL, USA; ^3^Department of Public Health Sciences, Miller School of Medicine, University of MiamiMiami, FL, USA

**Keywords:** Rift Valley fever, transmission dynamics, live vaccine, killed vaccine, seasonality forces

## Abstract

Rift Valley fever virus (RVFV) is an arthropod-borne viral pathogen that causes significant morbidity and mortality in small ruminants throughout Africa and the Middle East. Due to the sporadic and explosive nature of RVF outbreaks, vaccination has proved challenging to reduce RVFV infection in the ruminant population. Currently, there are two available types of vaccines, live and killed, in endemic areas. In this study, two mathematical models have been developed to explore the impact of live and killed vaccines on the transmission dynamics of RVFV. We demonstrate in general that vaccination helps reduce the severity of RVF outbreaks and that less delay in implementation and more vaccination attempts and effective vaccines can reduce the outbreak magnitude and the endemic number of RVFV. However, an introduction of a number of ruminants vaccinated by live vaccines in RVFV-free areas may cause an outbreak and RVFV may become endemic if there is sustained use of live vaccines. Other factors that are the important determinants of RVF outbreaks include: unsustained vaccination programs, recruitment of susceptible ruminants, and the seasonal abundance of mosquitoes.

## 1. Introduction

Rift Valley fever virus (RVFV) is an arthropod-borne viral pathogen belonging to the *Phlebovirus* genus in the *Bunyaviridae* family. It has been known to have a considerable effect on domesticated animals and humans in Africa and the Middle East. The virus was first detected in 1930 in Kenya. It was initially confined to Africa and Egypt, but later moved into the Middle East in 2000 (Abdo-Salem et al., 2011b). Infection with RVFV in animals is often associated with bloody diarrhea, necrotic hepatitis, hemorrhages, and abortions. Mortality due to the infection in some species of ruminants is nearly 100% in young animals and approximately 20–30% in adults (Evans et al., 2008; McElroy et al., 2009). In addition, the abortion rate of pregnant ruminants ranges from 40 to 100% during an outbreak. Susceptibility of ruminants to RVFV infection varies among species of ruminants, breeds, ages, and viral strains; for example, sheep are more susceptible than cattle while infected camels have as low as 2% mortality rate and only occasional abortions (Munyua et al., 2010; Smith et al., 2010). Humans infected with RVFV typically experience mild symptoms including fever, myalgia, and headache. However, 1–3% of these cases may develop severe encephalitis, renal failure, fatal hepatitis, and hemorrhagic fever (Näslund et al., 2009; Smith et al., 2010).

Transmission of RVFV among ruminants is primarily by vectors. Numerous species of mosquitoes are able to transmit RVFV but *Aedes* and *Culex* are considered the main vectors (Fontenille et al., 1998; Abdo-Salem et al., 2011b). Humans can become infected with RVFV by mosquito bites or by contact with or inhalation of aerosols during the handling or slaughtering of infected ruminants. RVF outbreaks are normally sporadic [outbreaks occur between 10 and 15 years or between 3 and 5 years in some endemic areas (Andriamandimby et al., 2010; Nderitu et al., 2011)]. Outbreaks are often linked to the coincidence of heavy rainfall and flooding events that allow large numbers of mosquitoes to emerge, the presence of susceptible livestock, along with the presence of RVFV (El-Rahim et al., 1999). RVFV is associated with two distinct transmission cycles: low-level enzootic and epizootic (Hollidge et al., 2010). During enzootic periods when there is non-excessive rainfall in East Africa, it is believed that RVFV is maintained through vertical transovarial transmission of floodwater *Aedes* species especially in areas with shallow depression habitats or dambos (Linthicum et al., 1985, 1999). High viremia caused by infected *Aedes* mosquitoes that emerge from flooding events in infected ruminants may allow the spillover of RVFV to secondary vectors such as *Culex* or *Anopheles* mosquitoes (Bird, 2012). However, vertical transovarial transmission is not currently present in the Middle East and West Africa. Factors associated with epizootics in West Africa and high rain forest zones of coastal and Central Africa remain unknown (El-Rahim et al., 1999; Martin et al., 2008).

Because of the high number of competent vectors of RVFV, the intensification of international trade of live animals that may introduce infected ruminants into non-endemic areas with high densities of susceptible livestock, and the unknown impact of climate change, several national and international agencies have issued warnings of the heightened risk of RVFV introduction (Ikegami and Makino, 2009; Pepin et al., 2010). Typically, preventive measures to control the spread of RVFV include disease surveillance, strategic vaccination of livestock, intensive vector control, restriction of animal movement, bans on animal importation from RVF-endemic countries, and increase of public awareness (Al-Afaleq and Hussein, 2011). Due to the severity of economic consequences from RVF outbreaks, routine immunization of lambs and calves is recommended. However, it is prohibitively expensive in Africa and sustaining vaccination programs in ruminants between outbreaks has proved difficult (Rusnak et al., 2011). Currently, two types of vaccines are available in endemic areas for the prophylactic immunization of ruminants (von Teichman et al., 2011).

Live attenuated RVFV vaccines (or live vaccines) provide long-term protective immunity without booster inoculations and are inexpensive to produce. The vaccines were developed from the Smithburn strain of RVFV by serial passages in mouse brains (Smithburn, 1949; Ikegami and Makino, 2009). As the neuroadapted virus only partially lost its virulence, this vaccine may induce abortions and teratogenesis in pregnant ruminants, and has the potential for reversion and capability to cause viraemia. Mosquitoes feeding on vaccinated ruminants may become infected and transmit RVFV to other ruminants and humans (Ikegami and Makino, 2009; Pepin et al., 2010; Kamal, 2011). Consequently, live vaccines are restricted and only used during devastating outbreaks. They should not be used in pregnant and young ruminants, and are not recommended in countries where RVFV has not been introduced. Moreover, the vaccines should not be administered to animals during breeding seasons of mosquitoes. According to vaccine description, animals used for human consumption should not be slaughtered within 21 days after vaccination. Used syringes, needles and remaining vaccine in bottles should be disposed hygienically (Kamal, 2011).

Formalin-inactivated RVFV vaccines (or killed vaccines) can be administered to animals of all ages and are safer than live vaccines. However, they are not as efficacious as live vaccines and thus repeated inoculations are required to induce and maintain protective immunity since an initial dose may only immunize a ruminant only for 6–12 months (Ikegami and Makino, 2009). Because these vaccines consist of relatively concentrated suspensions of the virulent virus that have been inactivated by formaldehyde or other chemical substances, they would be suitable for non-endemic areas and animals exported from endemic to RVFV-free areas (Wolrld Health Organization, 1982; von Teichman et al., 2011). Although killed vaccines have advantages of safety, they are costly to produce (Wolrld Health Organization, 1982).

Clearly, the use of live and killed vaccines to control the spread of RVFV is hampered by their disadvantages and highly effective vaccines are needed. New generation vaccines are currently under development and under clinical trials: (1) the attenuated MP12 which is derived from the virulent Egyptian strain (ZH548) and a plaque isolate of RVFV 74HB59, and (2) Clone 13 which is an avirulent candidate with no reversion owing to a large deletion in the NSs protein (that has been pointed out to be a virulence factor in animals), for instance (Ikegami and Makino, 2009; Pepin et al., 2010; Rusnak et al., 2011). Virus-like particle (VLP) approach and immunization with plasmids are examples of alternative approaches to develop vaccines (Ikegami and Makino, 2009; LaBeaud, 2010). Effective vaccines surely will facilitate the preparedness for prevention of an introduction of RVFV to disease-free areas and help reduce economic losses from dead and aborted ruminants. The ideal vaccine would be one that is safe without causing any pathogenic reaction and virulence reversion and confers long-term protection within a single dose. In addition, it should provide the differentiability between naturally infected and vaccinated animals (DIVA), and should not be expensive and difficult to produce (LaBeaud, 2010). Although vaccines can induce immunity against RVFV, it is important to recognize that recombination of live vaccinal strains and virulent strains is possible. Vaccines with deleted genes can reobtain those missing genes and cause serious consequences for disease elimination (Kamal, 2011).

Many modeling tools have been used to explore the risk of recurrent outbreaks in the endemic areas and the risk of RVF introduction in disease-free areas: climatic indices, spatial techniques, multi-variable statistical analysis, and dynamical transmission models (see Métras et al., 2011 for a review). Though there are some studies using dynamical transmission models (Bicout and Sabatier, 2004; Favier et al., 2006; Gaff et al., 2007; Mpeshe et al., 2011; Xue et al., 2012; Gao et al., 2013; Chamchod et al., 2014; Xiao et al., 2015), to the best of our knowledge, none of these papers has addressed the use of live and killed vaccines which is a clearly important and currently used means to control RVF epizootics and enzootics. For example, Bicout and Sabatier (2004) developed a stochastic model to investigate the prevalence of RVFV by including the seasonal abundance of mosquitoes. Favier et al. (2006) studied the endemicity of RVFV through vertical transmission in mosquitoes. In 2007, Gaff et al. (2007) developed a mathematical model by considering two species of mosquitoes. Apart from mosquito and livestock population, Mpeshe et al. (2011) incorporated a human population to investigate the prevalence of RVFV. In this study, we now develop two mathematical models to investigate the transmission dynamics of RVFV and the impacts of using live or killed vaccines. These two new models that capture advantages and disadvantages of live and killed vaccines incorporate several factors that may influence RVFV activity such as delay in vaccination, efficacy of vaccines, recruitment of animals, quarantine strategies, the abundance of mosquitoes, and vaccination strategies, in order to explore severity of RVF outbreaks, the prevalence of RVFV, the recurrence of outbreaks and the virus introduction. Our study provides an important insight into the effects of implementation of live or killed vaccines as an RVF control measure and underlines the need for effective vaccines. Our approach can also possibly be applied to explore certain diseases for which live or killed vaccines are used as preventive tools. West Nile virus, in which a number of candidates for live and killed vaccines are currently in various stages of testing, is a possible example (Tesh et al., 2002).

## 2. Methods

**Figure 1:**
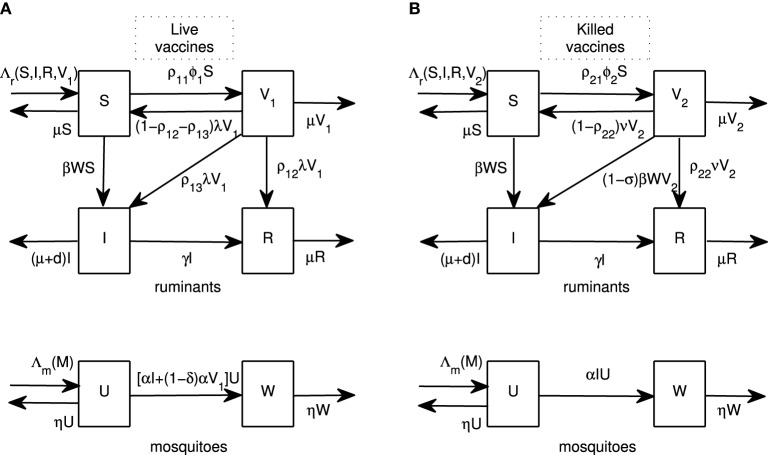
**Flow diagrams**. Flow diagrams for RVFV transmission between ruminants and mosquitoes for live and killed vaccines are shown in **(A,B)**, respectively. Ruminants are divided into four classes: susceptible (*S*), infectious (*I*), recovered (*R*), and vaccinated by live vaccines (*V*_1_) or killed vaccines (*V*_2_). Mosquitoes are divided into two classes: susceptible (*U*) and infectious (*W*).

We begin by introducing vaccination models for live and killed vaccines. A ruminant population at time *t* (*N*(*t*)) is divided into classes of susceptible (*S*(*t*)), infectious (*I*(*t*)), recovered (*R*(*t*)) and vaccinated by live vaccines (*V*_1_(*t*)) or vaccinated by killed vaccines (*V*_2_(*t*)) ruminants. A population of adult female mosquitoes at time *t* (*M*(*t*)) is divided into susceptible (*U*(*t*)) and infectious (*W*(*t*)) classes. Flow diagrams for both models are shown in Figure 1 and sample parameter values are shown in Table 1. To construct the models, we now lay out the assumptions for each type of vaccine.

**Table 1:** **Lists of parameters for Rift Valley fever virus transmission**.

**Description**	**Symbol**	**Sample value**	**References**
Natural death rate in ruminants (year^−1^)	μ	1/5.7-1/2	Majok et al., 1991
Birth rate in ruminants (year^−1^)	*b*	2.3	Majok et al., 1991
Recovery duration (year)	τ	8/365	Pepin et al., 2010
Probability of death due to RVFV in ruminants	*m*	0.3	Evans et al., 2008
Rate of recovery in ruminants (year^−1^)	γ	(1−*m*)(1∕τ)	
RVF-related death rate in ruminants (year^−1^)	*d*	*m*(1∕τ)	
The maximum number of ruminants (reflecting limited resources)	*N*^0^	100,000	Estimated
Crowding parameter of ruminants	*q*	(*b* − μ)∕*N*^0^	
Proportion of surviving newborns from infectious ruminants	*r*_1_	0.6	McElroy et al., 2009
Proportion of surviving newborns from ruminants vaccinated by live vaccines	*r*_2_	0.72	Kamal, 2011
Vaccination rate (year^−1^)	ϕ_1_, ϕ_2_	365/141	Métras et al., 2011
Probability that ruminants are vaccinated	ρ_11_, ρ_21_	0–1, 0.8	Marawan et al., 2012
Probability of successfully acquiring immunity from live vaccines	ρ_12_	0–1, 0.9	Niklasson et al., 1985; Papin et al., 2011
Probability of reversion of virulence of live vaccines	ρ_13_	0–0.2, 0.05	Davies, 2006; Nguku et al., 2010
Probability of receiving a repeated dose of killed vaccine	ρ_22_	0–1, 0.8	Marawan et al., 2012
Duration of viraemia in ruminants vaccinated by live vaccines (year)	λ	21/365	Kamal, 2011
Duration of protection from a primary dose of killed vaccine (year)	ν	5/12	Kamal, 2011
Biting rate (year^−1^)	*a*	256	
Probability of successful infection in ruminants	*p*_*r*_	0.14	Turell et al., 2008
Probability of successful infection in mosquitoes	*p*_*m*_	0.35	Turell et al., 2008
Birth rate in mosquitoes (year^−1^)	*g*	73	Dye, 1984; Hancock et al., 2009
Death rate of mosquitoes (year^−1^)	η	365/60	Reiskind et al., 1987
Maximum mosquito:ruminant ratio at	*k*_0_	0–10, 1.5	Gupta et al., 1994 (varying)
The maximum number of mosquitoes	*M*^0^	$k_{0}N^{0}$	
Crowding parameter of mosquitoes	*x*	(*g*−η)∕*M*^0^	
Reduction factor of transmission from ruminants vaccinated by live vaccines to mosquitoes	δ	0–1, 0.8	(varying)
Reduction factor of transmission in ruminants vaccinated by killed vaccines	σ	0–1, 0.8	(varying)

### 2.1. Live vaccines

#### 2.1.1. Host demography

Susceptible numbers of ruminants are increased by births at a rate Λ_*r*_(*S, I, R, V*_1_). We further assume that due to limited resources or human demands and abortions of ruminants from RVFV, Λ_*r*_ is described by the logistic term (*b* − *qN*)(*S* + *R*) + *r*_1_(*b* − *qN*)*I* + *r*_2_(*b* − *qN*)*V*_1_, where *b* is the birth rate of ruminants; *q* is a parameter reflecting the limited number of ruminants in an area; *r*_1_ is a proportion of surviving newborns from infected ruminants [since RVF infection can cause high abortion in pregnant ruminants (McElroy et al., 2009)]; and *r*_2_ is a proportion of surviving newborns from ruminants vaccinated with live vaccines [as live vaccines can cause abortion in early-stage pregnant ruminants (Kamal, 2011)]. Ruminant numbers decrease due to natural death and slaughter at a rate μ. RVF infection causes high mortality in ruminants (Evans et al., 2008) and only some animals recover with life-long immunity (Barnard, 1979; Paweska et al., 2005). Hence, ruminants die due to RVFV at a rate *d* and recover at a rate γ.

#### 2.1.2. Vector demography

We assume that mosquitoes die at a rate η and there is no vertical transovarial transmission so that mosquitoes are born disease-free at a rate Λ_*m*_ which is described by a logistic term (*g* − *xM*)*M*, where *g* is the birth rate of mosquitoes and *x* is a crowding parameter for mosquitoes. Note that vertical transmission is present in East Africa but not currently present in the Middle East and West Africa (El-Rahim et al., 1999; Martin et al., 2008). Since we are primarily interested in understanding the effects of RVF on ruminant populations and how those effects are influenced by the use of vaccines, we do not include vertical transmission in our study.

#### 2.1.3. Live vaccines

Although live vaccines induce early and long-term immunity, they may cause viraemia in ruminants and have a potential for virulence reversion. Hence, they are not recommended in non- endemic areas or during the breeding season of mosquitoes or during disease outbreaks (Ikegami and Makino, 2009; Kamal, 2011). Susceptible ruminants are vaccinated at a rate ρ_11_ϕ_1_, where 1∕ϕ_1_ is the time period that ruminants remain susceptible before being vaccinated and only a fraction ρ_11_ of ruminants is actually vaccinated. Vaccinated ruminants leave the vaccination class at a rate λ with a probability of ρ_12_ to successfully acquire a life-long immunity, a probability of ρ_13_ that reversion to virulence occurs, and a probability of 1 − ρ_12_ − ρ_13_ for vaccine failure.

#### 2.1.4. Transmission

Susceptible ruminants become infected at a rate β*WS*, where β is a per capita transmission rate from infectious mosquitoes to susceptible ruminants and it is a function of a per capita biting rate (*a*) and a probability of successful infection in ruminants (*p*_*r*_). Susceptible mosquitoes become infected from biting infectious ruminants at a rate α*UI*, where α is a per capita transmission rate from infectious ruminants to susceptible mosquitoes and it is a function of a per capita biting rate (*a*) and a probability of successful infection in mosquitoes (*p*_*m*_). Here, we assume that ruminants vaccinated by live vaccines can transmit RVFV to mosquitoes due to viraemia but the transmission is reduced by a factor δ from the rate of transmission from infectious ruminants. If δ = 1, there is no viraemia in vaccinated ruminants and if δ = 0, there is no reduction of viraemia in vaccinated ruminants as compared to infectious ruminants.

The changes in abundances of ruminants and mosquitoes over time can be described by a system of ordinary differential equations as follows:

(1) $$\begin{array}{l} \;\dot{S}(t)=\Lambda_{r}(S,I,R,V_{1})+(1-\rho_{12}-\rho_{13})\lambda V_{1}-\beta WS\\ \;-\rho_{11}\phi_{1}S-\mu S,\\ \;\dot{I}(t)=\beta WS+\rho_{13}\lambda V_{1}-(\mu +d+\gamma )I,\\ \;\dot{R}(t)=\gamma I+\rho_{12}\lambda V_{1}-\mu R,\\ \dot{V_{1}}(t)=\rho_{11}\phi_{1}S-(\mu +\lambda )V_{1},\\ \dot{U}(t)=\Lambda_{m}(M)-\alpha IU-(1-\delta )\alpha V_{1}U-\eta U,\\ \dot{W}(t)=\alpha IU+(1-\delta )\alpha V_{1}U-\eta W. \end{array}$$

Note that because the lifespan of ruminants and the lifespan of Aedes mosquitoes, which are important vectors of RVFV, are longer than the incubation period of RVFV, we simplify the models by not including the exposed classes in both animals.

### 2.2. Killed vaccines

#### 2.2.1. Host demography

As killed vaccines are safe and do not lead to abortions in ruminants (Ikegami and Makino, 2009; Kamal, 2011), we assume that Λ_*r*_ is described by the logistic term (*b* − *qN*)(*S* + *R* + *V*_2_) + *r*_1_(*b* − *qN*)*I*.

#### 2.2.2. Vector demography.

We use similar assumptions as live vaccines.

#### 2.2.3. Killed vaccines

Although killed vaccines are safer than live vaccines, they may have poor immunogenicity by not inducing long-term immunity and often requiring multiple vaccination doses (Ikegami and Makino, 2009; Bird, 2012). We assume that susceptible ruminants are vaccinated at rate ρ_21_ϕ_2_, where 1∕ϕ_2_ is the time period that ruminants remain susceptible before being vaccinated by killed vaccine and only a fraction ρ_21_ of ruminants is actually vaccinated. Vaccinated ruminants leave the vaccination class at rate ν with a probability of ρ_22_ to receive booster vaccines and successfully acquire long-term immunity, and a probability of 1 − ρ_22_ for individuals to become susceptible again due to vaccine failure or not receiving booster vaccines.

#### 2.2.4. Transmission

Not only susceptible ruminants but also ruminants vaccinated by killed vaccines [that may not induce complete protection against infections due to waning of an effective level of immunity (Boshra et al., 2011; Bird, 2012)] may become infected. However, we assume that infectiousness in the latter group of animals is reduced by a fraction σ, which represents the degree of protection induced by primary vaccination. Hence, susceptible and vaccinated ruminants become infected at rates β*WS* and (1 − σ)β*WV*_2_, respectively. Note that there is full protection against infections by killed vaccines if σ = 1 and there is no protection if σ = 0. Unlike live vaccines, killed vaccines with proper inactivation are not likely to cause viraemia in animals (Bird, 2012). Hence, we assume that only infectious ruminants can transmit RVFV to mosquitoes at rate α*UI*.

The assumptions lead to the following system of equations:

(2) $$\begin{array}{l} \;\dot{S}(t)=\Lambda_{r}(S,I,R,V_{2})+(1-\rho_{22})\nu V_{2}-\beta WS-\rho_{21}\phi_{2}S-\mu S,\\ \;\dot{I}(t)=\beta WS+(1-\sigma )\beta WV_{2}-(\mu +d+\gamma )I,\\ \;\dot{R}(t)=\gamma I+\rho_{22}\nu V_{2}-\mu R,\\ \dot{V_{2}}(t)=\rho_{21}\phi_{2}S-(1-\sigma )\beta WV_{2}-(\mu +\nu )V_{2},\\ \dot{U}(t)=\Lambda_{m}(M)-\alpha IU-\eta U,\\ \dot{W}(t)=\alpha IU-\eta W. \end{array}$$

### 2.3. Periodic vaccination, recruitment of animals, and seasonal variation

#### 2.3.1. Periodic vaccination

When vaccination attempts are periodic-like and not continuous, it is assumed that

$$\rho_{11}(t)=\rho_{21}(t)=\{\begin{array}{cc} 0.8, & i-1\leq t\leq i,\\ 0, & i\leq t\leq i+1, \end{array}\text{ where }i=1,3,5,\cdots ,$$

where *i* represents the vaccination period (that is, a year). This means that a contant vaccination rate (80%) is applied throughout the first year and no vaccination in the second year, followed by a contant vaccination in year 3 and no vaccination in year 4, and so on.

#### 2.3.2. Recruitment of animals

Ruminants are often recruited to replace dead or consumed animals in many areas. In this study, the pulse terms of animal recruitment are incorporated into the equation of Ṡ to represent an introduction of susceptible animals at particular time points as follows:

(3) $$\begin{array}{l} \overset{\infty }{\underset{n=1}{\sum }}\left(N^{0}-N\left(t\right)\right)\delta \left(t-nT\right), \end{array}$$

where *T* is a fixed period of introduction, *n* = 1, 2, 3, …, and δ is a Dirac delta function such that δ(*t* − *nT*) = 1 when *t* = *nT* and δ(*t* − *nT*) = 0 elsewhere. By adding the term (*N*^0^ − *N*(*t*))δ(*t* − *nT*) at every recruitment time, the total population always starts at *N*^0^ at each recruitment point. In case there is a variety in recruitment, (3) can be added in other disease classes and adjusted by multiplying it with percentages of recruited animals to be in each disease status.

#### 2.3.3. Seasonal variation of mosquitoes

To consider the effect of seasonal abundance of mosquitoes, we assumed that the mosquito:ruminant ratio (*k*) fluctuates over time as a sinusoidal function such that the mosquito:ruminant ratio is highest at the middle of each wet season and lowest at the middle of each dry season as follows:

$$k=k_{1}\left(1-k_{2}\cos2\pi t\right),$$

with *k*_2_ = (*k*_max_∕*k*_min_ − 1)∕(*k*_max_∕*k*_min_ + 1), *k*_1_ = *k*_max_∕(1 + *k*_2_), and *M* = *kN*^0^ (Altizer et al., 2006; Childs and Boots, 2010) with k ranging from 0.2 to 2 in our numerical study.

Note that the initial conditions unless they are varied for our numerical studies are as follows: *S*(0) = 99, 995, *I*(0) = 5, *R*(0) = 0, *V*_1_(0) = 0 = *V*_2_(0), *U*(0) = 150, 000 and *V*(0) = 0.

## 3. Results

### 3.1. The basic reproductive number

When vaccination is not implemented, both models (1) and (2) lead to the same basic reproductive number (*R*_0_). Without vaccination, RVFV dies out if *R*_0_ < 1 and is endemic if *R*_0_ > 1, where

(4) $$\begin{array}{l} R_{0}=\frac{\beta \alpha M^{0}N^{0}}{\eta \left(\mu +d+\gamma \right)} \end{array}$$

**Figure 2:**
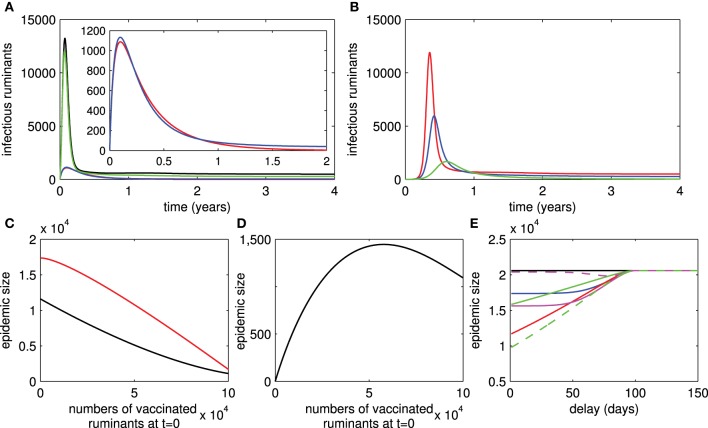
**Live and killed vaccines**. Infectious numbers of ruminants over time in case of live vaccines are shown in **(A)** (black trace = with no vaccination and *R*_0_ > 1, green trace = with vaccination and *R*_0_ > 1, blue trace = with vaccination and *R*_0_ < 1, red trace = with no vaccination and *R*_0_ < 1). Infectious numbers in case of killed vaccines are shown in **(B)** (red trace = with no vaccination and $R_{0}^{k}>1$, blue trace = with vaccination and $R_{0}^{k}>1$, green trace = with vaccination and $R_{0}^{k}<1$). **(C)** Shows that the epidemic size of an outbreak decreases when the number of vaccinated ruminants at *t* = 0 increases and *R*_0_ > 1 for both live and killed vaccines (red trace = live vaccines, black trace = killed vaccines). The epidemic size of an outbreak when the number of vaccinated ruminants at *t* = 0 varies and *R*_0_ < 1 for live vaccines is shown in **(D)**. **(E)** Shows the epidemic size of an outbreak corresponding to the delays in vaccination after an infectious ruminant is introduced for live and killed vaccines (red and solid traces: ρ_11_ = 0.8, ρ_12_ = 1, ρ_13_ = 0, δ = 0.9, magenta and solid traces: ρ_11_ = 0.8, ρ_12_ = 0.9, ρ_13_ = 0.05, δ = 0.8, magenta and dashed traces: ρ_11_ = 0.8, ρ_12_ = 0.8, ρ_13_ = 0.05, δ = 0.8, green and dashed traces: ρ_21_ = 0.8, ρ_22_ = 0.8, σ = 0.8, blue and solid traces: ρ_21_ = 0.8, ρ_22_ = 0.8, σ = 0.8, green and solid traces: ρ_21_ = 0.8, ρ_22_ = 1, σ = 0.9).

[see Section 1 in the Supplementary Material for the derivation of this formula and analysis of the system (1)]. From this formula, we can see that persistence of RVFV depends on transmission rates between ruminants and mosquitoes, numbers of ruminants and mosquitoes, lifespan of ruminants and mosquitoes, RVF-related death rate, and recovery rate. However, when live vaccines are used, RVFV always persists even when *R*_0_ < 1. Hence, we cannot find a formula for the basic reproductive number for the live-vaccine case. The persistence of RVFV when live vaccines are used is due to reversion to virulence and transmission during viremia of vaccinated ruminants. Figure 2A shows that RVFV is endemic when *R*_0_ > 1 regardless of whether vaccination is implemented. However, when *R*_0_ < 1, RVFV dies out when there is no implementation of live vaccines and it is endemic when there is implementation.

In case killed vaccines are used in an area, RVFV dies out if $R_{0}^{k}<1$ and is endemic if $R_{0}^{k}>1,$ where $R_{0}^{k}$ is the basic reproductive number when killed vaccines are used and is given by

(5) $$\begin{array}{l} R_{0}^{k}=R_{0}\frac{\mu \left(\mu +\nu \right)+\left(1-\sigma \right)\mu \rho_{21}\phi_{2}}{\left(\rho_{21}\rho_{22}\phi_{2}\nu +\rho_{21}\phi_{2}\mu +\mu \left(\mu +\nu \right)\right)}<R_{0} \end{array}$$

[see Section 2 in the Supplementary Material for the derivation of this formula and analysis of the system (2)]. Differently, persistence of RVFV in ruminant and mosquito populations depends on parameters associated with killed vaccines, vaccination rate, vaccine coverage, and the probability of receiving a repeated dose of killed vaccine. Consequently, it may be possible to eliminate RVFV by increasing vaccination attempts. In Figure 2B, whether killed vaccines are implemented, RVFV is endemic when $R_{0}>R_{0}^{k}>1$. However, by increasing vaccination attempts so that $R_{0}^{k}<1$, RVFV dies out.

Under the assumption that some ruminants are vaccinated by live or killed vaccines before an outbreak occurs (42% of the ruminant population for instance: this is quantity estimated from the vaccination rate and 2 months of advance notice of RVF activity in Table 1), the magnitude of an outbreak in our numerical studies (which we will call the epidemic size through the rest of this work) when live vaccines are used or ruminants vaccinated by live vaccines are introduced in areas is higher than when killed vaccines are used (Figures 2A,B). Note that there is a subtle difference when vaccination is not continued after an introduction of diseased or vaccinated ruminants. Moreover, in Figures 2A,B, it can be clearly seen that outbreaks in areas where killed vaccines are used occur in a later time than areas where live vaccines are used. We further investigated the duration from an introduction of diseased ruminants to the time that a RVF outbreak peaks (see Section 3 in the Supplementary Material). We found that this duration is shorter in areas where live vaccines are administered in comparison to areas where killed vaccines are used. This duration can be shortened by increasing the vaccine coverage and decreasing the vaccine efficacy. In contrast, the duration can be lengthened by increasing the probability that ruminants are vaccinated by killed vaccines and vaccine boosters. Hence, outbreaks may peak in areas where live vaccines with poor efficacy are heavily used before areas in which killed vaccines are effectively administered.

### 3.2. Initial control and delay

From the above results, the basic reproductive number can become a very useful measure to determine whether RVFV can spread among the ruminant and mosquito populations, and to investigate the severity of disease spread. The latter as measured by the epidemic size and endemic number Brauer and van den Driessche (2008) is an increasing function of the basic reproductive number. Note that the epidemic size is the maximum number of infectious ruminants during an outbreak and the endemic number is described by the number of infectious ruminants at the disease-present steady state. In addition, there are several factors that influence the severity of disease spread: the number of vaccinated ruminants at the beginning of an outbreak and the delays in implementing a vaccination control, for instance. Figure 2C shows that the epidemic size is reduced by increasing the initial number of vaccinated ruminants by live or killed vaccines. As it cannot be easily seen in Figure 2C, we further investigated tangent slopes that represent the changes of epidemic sizes with respect to the changes of numbers of ruminants vaccinated by live or killed vaccines. We found that (1) the reduction of epidemic sizes occurs slowly when fewer numbers of ruminants are vaccinated by live vaccines and increases quickly when vaccinated numbers become larger, and (2) the reduction occurs quickly when fewer numbers of ruminants are vaccinated by killed vaccines and moderately when larger numbers of ruminants are vaccinated. Since ruminants vaccinated by live vaccines may transmit RVFV to mosquitoes, we investigated whether an outbreak occurs when ruminants are vaccinated by live vaccines as a preparedness plan or introduced in areas with *R*_0_ < 1 and no vaccination. Figure 2D shows that an outbreak occurs and vaccinated numbers have a negative impact on the epidemic size when they increase. However, when the number of ruminants vaccinated by live vaccines is approximately more than a half of the population size, the epidemic size starts to decrease. In Figure 2E, when there is a delay in vaccination by live or killed vaccines, the longer the delay is, the larger the epidemic size gets. The epidemic size eventually reaches the same size with when vaccination is not implemented if the delay is sufficiently long enough (approximately 3 months).

### 3.3. The epidemic size and endemic number

**Figure 3:**
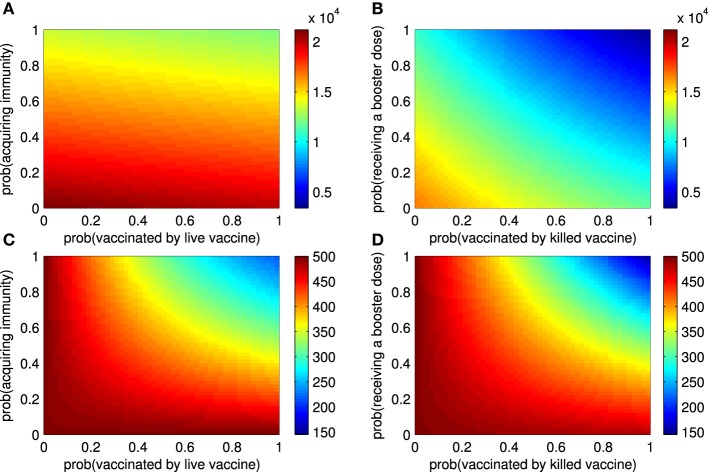
**The epidemic size and endemic numbers. (A)** The epidemic size of an outbreak for live vaccines when the probability that ruminants are vaccinated (ρ_11_) and the probability that they acquire immunity (ρ_12_) vary. **(B)** The epidemic size of an outbreak for killed vaccines when the probability that ruminants are vaccinated (ρ_21_) and the probability that they receive repeated doses later (ρ_22_) vary. **(C)** The endemic number of RVFV among ruminants changes according to ρ_11_ and ρ_12_. **(D)** The endemic number of RVFV among ruminants changes according to ρ_21_ and ρ_22_.

Next, we investigated the impact of vaccination attempts (ρ_11_, ρ_21_, ρ_22_) and vaccine efficacy (ρ_12_) on the epidemic size and endemic number. Figure 3A shows that the probability that ruminants are vaccinated by live vaccines has a small effect on the epidemic size as compared to the probability of successfully acquiring immunity of ruminants. Both have minute effects when no ruminants are vaccinated by live vaccines at the beginning of an outbreak (Section 3 in the Supplementary Material). When some ruminants are vaccinated by killed vaccines before an outbreak starts, the probability that ruminants are vaccinated by killed vaccines and probability that ruminants receive repeated doses influence the epidemic size by decreasing it. In case none of the ruminants are vaccinated by killed vaccines before an outbreak, the former has a greater impact than the latter when the vaccines are administered later (Figure 3B and Section 3 in the Supplementary Material). As shown in Figures 3C,D, all of those vaccines quantities have an impact on the endemic number of RVFV in ruminants. By further investigating the epidemic size and endemic number with other vaccine efficacy terms (ρ_13_, δ, and σ), the results lead to a similar conclusion that the better the vaccine efficacy is, the smaller the epidemic size and endemic number are. Note that additional results not included in Figure 3 can be found in Section 3 in the Supplementary Material.

### 3.4. Periodic vaccination

**Figure 4:**
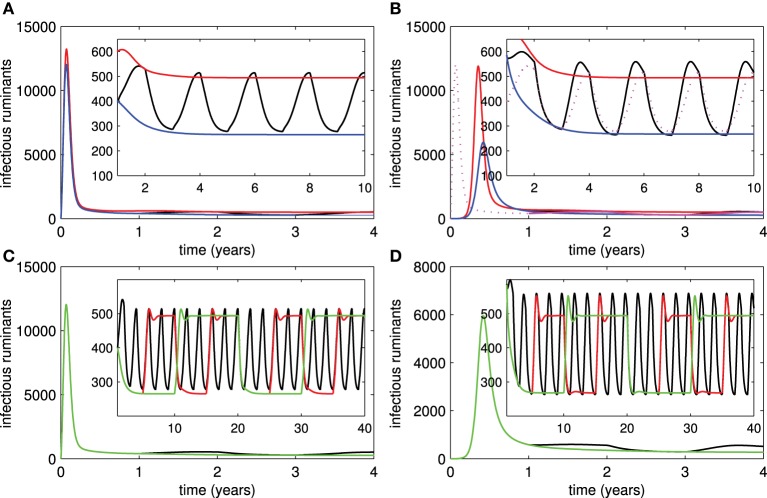
**Periodic vaccination**. **(A,B)** Show numbers of infectious ruminants when periodic vaccination of live and killed vaccines is implemented, respectively (red trace = no vaccination, blue trace = constant vaccination, black trace = periodic vaccination for every 1 year). **(C,D)** Show numbers of infectious ruminants according to periodic vaccination of live and killed vaccines when vaccination periods change, respectively (black trace = 1-year period, red trace = 3-year period, green trace = 5-year period).

Because of the periodic or sporadic nature of RVF outbreaks and economically limited access to vaccines, sustained vaccination efforts on RVFV have proved difficult in many endemic areas. Figures 4A,B show the number of infectious ruminants in correspondence with the periodic vaccination of live or killed vaccines for every two years. From the results, the periodic vaccination efforts may cause small outbreaks in both live and killed vaccine cases. Although the first outbreak is more serious when live vaccines are used, it is more likely that subsequent outbreaks are smaller than when killed vaccines are used. On the other hand, killed vaccines have a greater impact on reducing the severity of outbreaks if the vaccination period is lengthened as compared to live vaccines (Figures 4C,D).

### 3.5. Recruitment of animals

**Figure 5:**
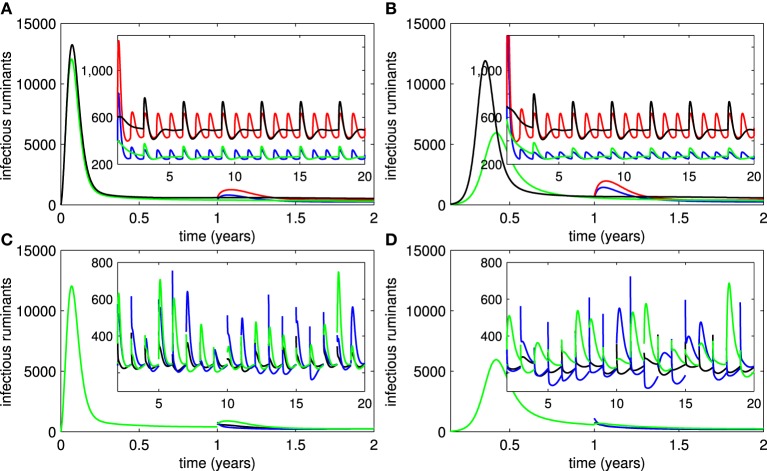
**Recruitment of ruminants**. **(A,B)** Show the numbers of infectious ruminants corresponding to introduction of susceptible ruminants into areas where live or killed vaccines are used, respectively (red trace = with every year recruitment and no vaccination, black trace = with every three year recruitment and no vaccination, blue trace = with vaccination and every 3 recruitment, green trace = with vaccination and every 3 year recruitment). **(C,D)** Show the number of infectious ruminants corresponding to consumption and introduction of ruminants in different disease statuses for live and killed vaccines, respectively (black trace = fewer numbers of consumed ruminants and larger numbers of recruited ruminants with immunity, blue trace = larger numbers of consumed ruminants and recruited ruminants with immunity, green trace = fewer numbers of consumed ruminants and recruited ruminants with immunity).

We investigated the introduction of new susceptible ruminants into the ruminant population at the beginning of every year or every three years (these periods can be adjusted to account for the banning of imported animals by the government after an outbreak occurs) by introducing pulse functions (3) of animal recruitment (1) and (2). Figures 5A,B suggest that small outbreaks occur in areas in which ruminants are recruited. Their frequency is reduced by an extended period of animal recruitment and their severity is decreased by implementing vaccination (live or killed). Let us assume further that some ruminants in the endemic areas are consumed at the end of every year for a religious feast and a variety of new ruminants are recruited to replace them and other dead ruminants afterward. In Figures 5C,D, we assumed that (1) 20 or 50% of ruminants are eaten during a feast in each year, (2) more than 80 or 50% of recruited ruminants are immune to RVFV (ruminants are vaccinated and quarantined until they successfully acquire immunity before the recruitment), (3) less than 1% of recruited ruminants are infectious, and (4) other recruited ruminants are either susceptible or vaccinated. The probability that ruminants are in each disease status was chosen randomly in our simulation study and only the lower bound of the percentage of immune ruminants and upper bound of the percentage of infectious ruminants in recruited animals were given. Hence, for instance,

$$-0.2\overset{\infty }{\underset{n=1}{\sum }}R\left(t\right)\delta \left(t-n\left(T-\varepsilon \right)\right)+p_{\geq 0.8}^{random}\overset{\infty }{\underset{n=1}{\sum }}\left(N^{0}-N\left(t\right)\right)\delta \left(t-nT\right),$$

where ε → 0 (20% of immune ruminants are consumed and more than 80% of recruited ruminants are immune), was added into the equation for *Ṙ*. We found that RVF outbreaks are more likely to occur when the percentage of recruited ruminants with immunity to RVFV is reduced and when more ruminants are consumed during the feast. The percentage of recruited ruminants with acquired immunity has a significant impact on both frequency and severity of outbreaks more than the number of consumed ruminants. Moreover, outbreaks are more severe when live vaccines are used in endemic areas (spiny peaks for live vaccines and unpointed peaks for killed vaccines).

### 3.6. Mosquito activity

**Figure 6:**
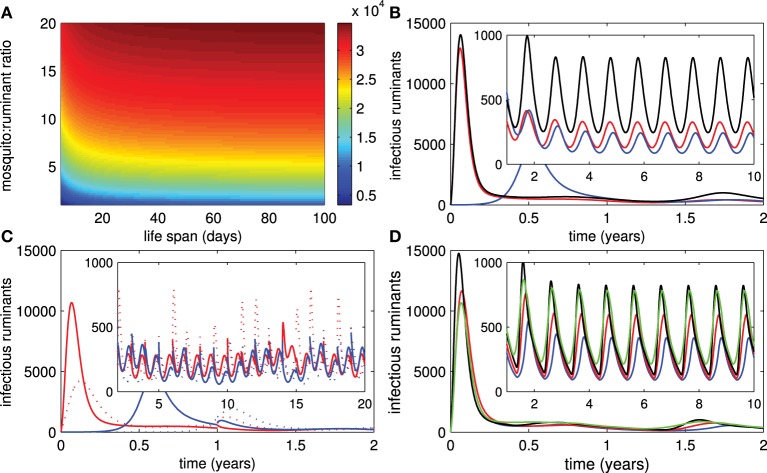
**Mosquito activity. (A)** The epidemic size of an outbreak according to changes in the mosquito lifespan and the mosquito:ruminant ratio. **(B)** The number of infectious ruminants changes according to the seasonal abundance of mosquitoes. **(C)** The changes in infectious numbers of ruminants relating to the yearly introduction of ruminants with different disease statuses during high and low levels of mosquito activity for live and killed vaccines (red and solid trace = introduction of ruminants during a low level of mosquito activity for live vaccines, red and dotted trace = introduction of ruminants during a high level of mosquito activity for live vaccines, blue and solid trace = introduction of ruminants during a low level of mosquito activity for killed vaccines, blue and dotted trace = introduction of ruminants during a high level of mosquito activity for killed vaccines). **(D)** The changes in infectious numbers relating to seasonal vaccination of live vaccines when the mosquito:rumiant ratio peaks during a rainy season (*k*_0_ = 2) and is less abundant in other seasons (*k*_0_ = 1) (blue trace = vaccination is not implemented in a rainy season which lasts 4 months, red trace = vaccination is implemented in a rainy season which lasts 4 months, green trace = vaccination is not implemented during a rainy season that lasts 6 months, black trace = vaccination is not implemented in the areas).

It has been suggested that RVF outbreaks are often associated with high numbers of mosquitoes. Figure 6A shows that the mosquito:ruminant ratio has a drastic effect (as compared to the mosquito lifespan) on the epidemic size so that a higher mosquito:ruminant ratio leads to a larger epidemic size of the outbreak. Hence, when the seasonal abundance of mosquitoes is taken into account, after the first outbreak the number of infectious ruminants peaks slightly after the middle of each year that the mosquito:ruminant ratio is highest (see Figure 6B). Next we assumed that 20% of ruminants are consumed at the end of each year and replaced ruminants are recruited with at least 50% of them immune to RVFV and less than 1% of them infectious. Figure 6C shows that (1) recruiting ruminants during the high activity instead of low activity of mosquitoes may cause outbreaks, and (2) serious outbreaks are more likely to occur in areas where live vaccines are administered and larger numbers of ruminants with no immunity are recruited. In Figure 6D, small outbreaks occur when ruminants are vaccinated by live vaccines during seasons with high activity of mosquitoes. However, when seasons with high activity of mosquitoes are lengthened, the severity of those outbreaks increases and implementation of live vaccines is recommended even during seasons with high activity of mosquitoes. This result is not surprising because routine and continuous vaccination is probably better than having no vaccination.

## 4. Discussion

We have developed two mathematical models to investigate the transmission dynamics of RVFV among ruminants via mosquitoes and the impacts of using live or killed vaccines to control the spread of RVFV. Advantages and disadvantages of live and killed vaccines were taken into account. Several factors that may influence the severity and recurrence of RVF outbreaks such as delay in vaccination, vaccination efforts, vaccine efficacy, recruitment of animals, quarantine strategies, the seasonal abundance of mosquitoes, and vaccination strategies were considered in our study.

### 4.1. The basic reproductive number

It has been observed in many endemic areas that the prevalence of RVFV remains at a very low level after an outbreak: 1–3% in certain areas of Africa during a non-epizootic period and as low as 0.1% in Yemen (Davies et al., 1992; Rostal et al., 2010; Abdo-Salem et al., 2011b). Similarly, our models predicted that RVFV remains endemic at a very low level after an outbreak since large numbers of infected ruminants become immune to RVFV. The basic reproductive number (*R*_0_) is an important quantity in epidemiology that has played a crucial role in disease control. It potentially determines whether a disease can spread through a population and is defined as the expected number of secondary infections resulting from an introduction of a single infected individual into a completely susceptible population. The number of infected individuals increases if *R*_0_ > 1 and decreases if *R*_0_ < 1 (Brauer and van den Driessche, 2008). It also helps determine persistence and severity of the disease spread as the epidemic size and the endemic number of infected individuals are increasing functions of it. To eliminate the parasite or reduce its severity and burden, sustained disease control needs to be implemented to ensure that *R*_0_ is less than one or as small as possible. Our results demonstrate that the basic reproductive number of RVFV without vaccination generally depends on transmission rates between ruminants and mosquitoes, numbers of ruminants and mosquitoes, lifespan of ruminants, RVF-related death rate, and recovery rate and is given by

$$R_{0}=\frac{\beta \alpha M^{0}N^{0}}{\eta \left(\mu +d+\gamma \right)}.$$

In case live vaccines are constantly administered, RVFV persists despite *R*_0_ < 1 (due to the possibility that ruminants vaccinated by live vaccines may transmit RVFV to mosquitoes and reversion to virulence of live vaccines in ruminants may occur) and there is no particular formula for the basic reproductive number that gives information of live vaccines. Contrarily, the basic reproductive number when killed vaccines are used as a preventive tool $\left(R_{0}^{k}\right)$ can be calculated ($R_{0}^{k}<R_{0}$) and is associated with the killed vaccine parameters. This suggests that it may be possible to eliminate RVFV by increasing a vaccination attempt on killed vaccines so that $R_{0}^{k}<1$. We further found that the magnitude of an outbreak or the epidemic size when live vaccines are used in prevention strategies is more likely to be higher than killed vaccines under the same vaccination rate. This may result from the possibility that vaccinated ruminants may transmit RVFV to mosquitoes and the possibility of reversion to virulence of live vaccines. Interestingly, the number of vaccinated ruminants and vaccine efficacy play an important role in the outbreak timing. The duration from an introduction of diseased ruminants to the time that the virus activity peaks is shortened by the larger number of ruminants vaccinated by live vaccines and poor efficacy of live vaccines. However, it is lengthened by increasing the number of vaccinated by killed vaccines and repeated doses. Knowing the period over which the outbreak extends could be useful in designing the effective mosquito control strategies. So far our results support several studies that suggest the use of killed vaccines in non-endemic areas and agree with studies that suggest the persistence of RVFV in areas that live vaccines are used owing to the contamination in environment and transmission of RVFV from vaccinated animals to mosquitoes (Kamal, 2009, 2011; von Teichman et al., 2011).

### 4.2. Initial control and delay

Although the basic reproductive number provides important information for the spread of RVFV, it only gives partial information on the severity of outbreaks (expressed via the epidemic size) and the endemic number. The number of vaccinated ruminants is one of the factors that plays a crucial role in reducing the number of infected ruminants during epizootic and enzootic cycles of RVFV. Remote sensing satellite data of sea-surface temperatures, rainfall, and intensity of green vegetation have been used to investigate and predict mosquito and RVF activity (Linthicum et al., 1999). Prediction from such information could provide a 2–6 week warning (Anyamba et al., 2009). Our results show that the epidemic size is reduced when more ruminants are vaccinated before an outbreak occurs. Hence, a better advance warning of RVF outbreaks may aid the control preparedness and provide sufficient time to vaccinate ruminants to raise the herd immunity, and consequently reduce the epidemic size of an outbreak. Moreover, we found that the reduction of epidemic sizes occurs slowly when fewer ruminants are vaccinated by live vaccines but changes dramatically when numbers of vaccinated ruminants become larger. The reduction occurs quickly when fewer ruminants are vaccinated by killed vaccines and changes moderately when more ruminants are vaccinated.

One of the surprising results obtained in our study is that an outbreak may occur in areas where ruminants are vaccinated by live vaccines for advance preparedness or introduced in areas with *R*_0_ < 1. Note that in both cases there is no further vaccination of live vaccines. The reason is that vaccinated ruminants may transmit RVFV to mosquitoes and cause an outbreak. Intermediate numbers of vaccinated ruminants may lead to a more serious outbreak than when a small or large number of ruminants are vaccinated. The results also suggest a possible trade-off between transmission of RVFV in vaccinated ruminants and herd immunity. Our finding supports several studies that suggest the use of killed vaccines in non-endemic areas (Kamal, 2009, 2011; von Teichman et al., 2011). The early detection of RVFV activities is an important key for the effective disease control to minimize outbreak consequences. However, it is possible that the delays in diagnosis and reporting infection cases may occur in several weeks or months between the presumptive start of an outbreak and its initial detection by public health and veterinary authorities (McElroy et al., 2009; Bird, 2012). Consequently, such delays may lead to the delay in launching a vaccination program.

Our results demonstrate that the delay in implementing vaccination can lead to more serious outbreaks and that outbreaks reach the same epidemic size with when no vaccination is implemented if the delay is long enough. A good example which supports this finding is a major 2006–2007 outbreak in East Africa in which the public health community was alerted for several months before the first confirmed human cases were reported. Few preventive steps were started before laboratory confirmation and there was a delay of almost 8 weeks in the administration of vaccines and subsequently that caused a substantial loss of humans and livestock (Anyamba et al., 2009; Bird, 2012). In addition, our results suggest that the delay has an effect on the epidemic size even though it is small for killed vaccines. In the case of live vaccines, the delay has a small effect when it is small but a larger effect when it becomes bigger. Hence, according to our findings, RVF outbreaks in non-endemic areas can be very drastic since there is presumably a delay in vaccination.

### 4.3. The epidemic size and endemic number

Vaccination attempts and vaccine efficacy are among the important determinants of effective control. Imperfect vaccines that give incomplete protection and the loss of vaccine-induced immunity, for instance, may not help prevent severe outbreaks and their resurgence efficiently (Keeling and Rohani, 2007). Our results suggest that inefficient vaccines and few vaccination attempts may lead to the magnified epidemic size and the larger endemic number. Furthermore, the percentage of ruminants vaccinated by live vaccines has a small impact on the epidemic size if the percentage of ruminants that successfully acquire long-term immunity is small. For killed vaccines, both of the percentage of vaccinated ruminants and the percentage of ruminants receiving boosters have an impact on the epidemic size and endemic number, but the former has a bigger impact when none of ruminants are vaccinated before an outbreak. Based on our findings, several factors also have a significant impact on the epidemic size and endemic number so that the better efficacy of vaccines may result in the smaller epidemic size and lower endemic number: the probability of reversion to virulence, the reduction factor of transmission from ruminants vaccinated by live vaccines to mosquitoes, and the reduced factor of infection in ruminants vaccinated by killed vaccines. Our results underline how important vaccination attempts and vaccine efficacy are for controlling RVFV and highlight the need of effective vaccines and vaccination strategies.

### 4.4. Periodic vaccination

Vaccination is an effective means to control the spread of RVFV and prevent disease-related losses but has proved challenging for RVFV due to the sporadic and explosive nature of RVF outbreaks and limited access to vaccines (McElroy et al., 2009). Sustaining vaccination programs for ruminants during enzootic cycles and mass vaccination of ruminants during ongoing epizootics are not normally possible (McElroy et al., 2009; Rusnak et al., 2011). We investigated the consequences of this periodic-like vaccination and found that the lack of continuous vaccination efforts may cause small outbreaks in endemic areas for both live and killed vaccines. Moreover, in the long term, subsequent outbreaks are smaller when live vaccines are used in comparison to killed vaccines. The use of killed vaccines may be better for reducing the severity of outbreaks when periods of using and discontinuing vaccines are lengthened as compared to when such periods are shortened. However, the lengths of such periods have less effects on live vaccines.

### 4.5. Recruitment of animals

Recruitment of ruminants into an area for consumption or maintenance of the herd sizes may involve the introduction of a massive number of susceptible ruminants. We investigated the impact of the presence of susceptible ruminants on epizootic and enzootic cycles and found that outbreaks may occur in the endemic areas when susceptible ruminants are recruited. However, the frequency of outbreaks can be reduced by the extended period of animal recruitment and their severity can be decreased by live or killed vaccine administration. This finding corresponds to some studies suggesting that cattle of owners who purchased ruminants to replace their herds following outbreaks were significantly more antibody-positive than others (Chevalier et al., 2011), and supports a control measure that suggest a ban of animal importation after an outbreak (Abdo-Salem et al., 2011b; Al-Afaleq and Hussein, 2011). In many areas, ruminants are imported and consumed for religious festivals (or are imported to replace dead or consumed ruminants) (Thiongane et al., 1997; Abdo-Salem et al., 2011b; Al-Afaleq and Hussein, 2011).

For international trade of ruminants, surveillance and certification systems are required for exported countries in order to minimize the risk of introduction of diseases; for example, Ethiopia has collection and quarantine points where ruminants are gathered, fed, treated, vaccinated, and kept for approximately 20–30 days (Abdo-Salem et al., 2011b). Since the importation of ruminants may involve a number of ruminants in different disease statuses, we explored how different percentages of immune (quarantined and vaccinated) and susceptible (quarantined but not vaccinated) ruminants and different percentages of animal consumption in the imported areas in which live or killed vaccines are used influence RVF activity.

Our results demonstrate that RVF outbreaks are more likely to occur when the percentage of recruited ruminants immune to RVFV is reduced and larger numbers of ruminants are consumed. More interestingly, the percentage of recruited ruminants with acquired immunity has a greater impact on both frequency and severity of outbreaks in comparison to the percentage of consumed ruminants. Further outbreaks are more severe in areas where live vaccines are used. These findings highlight the impact of animal recruitment and how important it is to immunize animals. They may provide important insights to design control strategies for importation of animals.

### 4.6. Mosquito activity

It has been observed in many studies that RVF outbreaks are closely linked to heavy rainfall and high numbers of mosquitoes (Linthicum et al., 1999; Anyamba et al., 2009; Bird, 2012). Furthermore, RVF virus activities often occur annually and are associated with seasonal rainfall during non-epidemic periods (Davies et al., 1992). Our results also show that (1) a larger number of mosquitoes may increase the epidemic size of an outbreak, (2) the number of mosquitoes has a higher impact on the epidemic size than the mosquito lifespan, and (3) RVF cases fluctuate according to the seasonal abundance of mosquitoes (with a small delay after a peak of mosquito numbers). Furthermore, one of the interesting results related to mosquito activities suggest that recruiting ruminants during a period with a high level of mosquito activity may promote a severe outbreak and serious outbreaks are more likely to occur in areas in which live vaccines are administered and many ruminants with no immunity are recruited. Though the effect of rainfall was not directly modeled and simulated in this paper, in another study our team (Gil et al., 2016) modeled the climate hydrology (rainfall) by using a sinusoidal carrying capacity for mosquitoes synched to a solar period, and found that concurrently high ruminant density and mosquito growth (with strong rainy season) can increase the likelihood of an RVF epizootic.

The findings may simultaneously result from the increased mosquito:ruminant ratio, larger numbers of susceptible ruminants, and transmission possibility of ruminants vaccinated by live vaccines. However, vaccination by live vaccines is recommended if the period with a high level of mosquito activity is annually longer than the period with a low level of mosquito activity. All in all, our results agree with the live vaccine description so that ruminants should not be vaccinated during breeding seasons of mosquitoes (Kamal, 2011) and may also link to some studies that suggest the emergence of RVF in Yemen in 2000 as the confluence of environmental conditions favorable to mosquitoes and high densities of imported ruminants for a religious feast (Abdo-Salem et al., 2011a). Based on our results implementing stringent control measures in imported ruminants during a mosquito season may help reduce the epizootic risk.

Together with several factors that may influence RVF activity, our models that capture advantages and disadvantages of live and killed vaccines allow us to investigate the impact of vaccination types on the RVFV transmission dynamics during epizootic and enzootic periods that (to our knowledge) have not been addressed in any previous modeling studies. Although some assumptions are made and there is limited information on certain model parameters (for example, the percentage of ruminants that receive booster vaccines was set high while it may be lower in real events), several findings in our study correspond to previous empirical studies while others make predictions that can be further investigated empirically. Finally, due to the complexity of RVF dynamics and several other factors, we did not use our models to fit the documented data from any particular region. However, we believe that this study may be useful in understanding the dynamics of RVFV among ruminants in areas in which vaccination is implemented, identifying the key variables, helping indicate advantages and disadvantages of live and killed vaccines, underlining the need of effective vaccines, and providing important insights to help design effective control strategies.

Since Aedes mosquitoes which are important vectors of RVFV have a longer lifespan (60–90 days) in comparison to Anopheles mosquitoes (14–30 days) that are important vectors to other diseases such malaria, and the incubation of RVFV in mosquitoes is around 2–4 days, this leads to a short period of mosquitoes staying exposed as compared to their lifespan. The probability that mosquitoes survive this exposed period is *e*^−ητ^, where η is the death rate of mosquitoes and τ is the incubation period in mosquitoes. This gives us *e*^−1∕30^ which is close to 1. On one hand, this term can be factored into the coefficient as β′ = β*e*^−ητ^. Since *e*^−1∕30^ is approximately equal to 1, β′ can be approximated by β. On the other hand, a more general way to model the exposed class is using a term β*e*^−ητ^*S*(*t* − τ)*W*(*t* − τ) in the models. Since τ is very small, the term can be possibly approximated by β*S*(*t*)*W*(*t*). Hence, we decided to not include the exposed class explicitly in our models since we mainly focused on modeling the effects of live and killed vaccines on the transmission dynamics of RVFV.

### Conflict of interest statement

The authors declare that the research was conducted in the absence of any commercial or financial relationships that could be construed as a potential conflict of interest.
