# Supplementary material for: Transmission Dynamics of Rift Valley Fever Virus: Effects of Live and Killed Vaccines on Epizootic Outbreaks and Enzootic Maintenance
Source: Front Microbiol. 2016 Feb 1;6:1568. doi: 10.3389/fmicb.2015.01568 (PMC4740790; doi:10.3389/fmicb.2015.01568)
Supplement: Supplementary file 1 [file Presentation1.PDF]

frontiers

Supplementary Material: Transmission dynamics of Rift Valley fever virus: effects of live and killed vaccines on epizootic outbreaks and enzootic maintenance

Farida Chamchod[1], Chris Cosner[2], Robert Stephen Cantrell[2], John C. Beier[3] and Shigui Ruan[2,*]

[1] *Department of Mathematics, Faculty of Science, Mahidol University, Bangkok 10400, Thailand*
[2] *Department of Mathematics, University of Miami, Coral Gables, FL 3324-4250, USA*
[3] *Department of Public Health Sciences, Miller School of Medicine, University of Miami, Miami, FL 33136, USA*

Correspondence*:
Shigui Ruan
Department of Mathematics, University of Miami, Coral Gables, FL 3324-4250, USA, ruan@math.miami.edu

1 ANALYSIS OF THE VACCINATION MODEL WITH LIVE VACCINES

Let us consider the following system of ordinary differential equations:

$$\begin{aligned}
\dot{S} &= \Lambda_r(S, I, R, V_1) + (1-\rho_{12}-\rho_{13})\lambda V_1 - \beta W S - \rho_{11}\phi_1 S - \mu S,\\
\dot{I} &= \beta W S + \rho_{13}\lambda V_1 - (\mu + d + \gamma) I,\\
\dot{R} &= \gamma I + \rho_{12}\lambda V_1 - \mu R,\\
\dot{V_1} &= \rho_{11}\phi_1 S - (\mu+\lambda) V_1,\\
\dot{U} &= \Lambda_m(M) - \alpha I U - (1-\delta)\alpha V_1 U - \eta U,\\
\dot{W} &= \alpha I U + (1-\delta)\alpha V_1 U - \eta W
\end{aligned} \tag{1}$$

with $\Lambda_r(S, I, R, V_1) = (b - qN)(S + R) + r_1(b - qN)I + r_2(b - qN)V_1$, $\Lambda_m(M) = (g - xM)M$, $q = (b - \mu)/N^0$ (where N^0 is the carrying capacity of the ruminant population), and $x = (g - \eta)/M^0$ (where M^0 is the carrying capacity of the mosquito population). Note that we always assume that $b > \mu$ and $g > \eta$ throughout our work for positive numbers of the maximum sizes of ruminant and mosquito populations, respectively. Following the properties of solutions of system (1), we define the closed and positively invariant set:

$$\Gamma = \left\{ (S, I, R, V_1, U, W) \in \mathcal{R}^6_+ \middle| \begin{array}{l} S + I + R + V_1 \le \frac{(b-\mu)}{q}, U + W \le \frac{(g-\eta)}{x}, \\ S \ge 0, I \ge 0, R \ge 0, V_1 \ge 0, U \ge 0, W \ge 0 \end{array} \right\}.$$

1.1 WITH VACCINATED RUMINANTS AT THE INTRODUCTION BUT NO ADMINISTRATION OF VACCINES AFTERWARD ($\rho_{11} = 0$ AND $V_1(0) \geq 0$)

Under the assumption that $\rho_{11} = 0$, the number of vaccinated ruminants by live vaccines is described as follows:

$$V_1(t) = V_1(0)e^{-(\mu+\lambda)t}. \tag{2}$$

It depends on the initial number of vaccinated ruminants and duration that ruminants stay in the vaccination class, and declines from $V_1(0)$ toward zero over time.

1.1.1 Steady states There are four disease-free steady states: $(S, I, R, V_1, U, W) = (0, 0, 0, 0, 0, 0), ((b - \mu)/q, 0, 0, 0, 0, 0), (0, 0, 0, 0, (g - \eta)/x, 0)$, and $((b - \mu)/q, 0, 0, 0, (g - \eta)/x, 0)$. The first three steady states are always unstable which can be proved by considering the signs of eigenvalues of the Jacobian matrix of (1) at each steady state or using Routh-Hurwitz stability criterion. Stability of the disease-free steady state $P^0 = ((b - \mu)/q, 0, 0, 0, (g - \eta)/x, 0)$ depends on the basic reproductive number of (1) and is further investigated below. There exists a disease-present (endemic) steady state without vaccination (P^*) in Γ: $(S, I, R, V_1, U, W) = (S^*, I^*, R^*, V_1^*, U^*, W^*)$ with

$$S^* = \frac{x(\mu + d + \gamma)(\eta + I^*)}{\beta(g - \eta)}, R^* = \frac{\gamma I^*}{\mu}, V_1^* = 0, U^* = \frac{\eta(g - \eta)}{\alpha x(\eta + I^*)}, W^* = \frac{(g - \eta)I^*}{x(\eta + I^*)},$$

and I^* satisfying the following second-order polynomial equation:

$$C_2 I^{*2} + C_1 I^* + C_0 = 0,$$

where

$$\begin{aligned} C_2 &= \left(\tfrac{x(\mu+d+\gamma)}{\beta(g-\eta)} + \tfrac{\gamma}{\mu}\right)\left(\tfrac{qx(\mu+d+\gamma)}{\beta(g-\eta)} + q + \tfrac{\gamma}{\mu}\right) + r_1 q\left(\tfrac{x(\mu+d+\gamma)}{\beta(g-\eta)} + 1 + \tfrac{\gamma}{\mu}\right), \\ C_1 &= \tfrac{x(\mu+d+\gamma)\eta}{\beta(g-\eta)}\left(\tfrac{qx(\mu+d+\gamma)}{\beta(g-\eta)} + q + \tfrac{\gamma}{\mu}\right) - \left(\tfrac{x(\mu+d+\gamma)}{\beta(g-\eta)} + \tfrac{\gamma}{\mu}\right)\left(b - \tfrac{qx(\mu+d+\gamma)\eta}{\beta(g-\eta)}\right) \\ &\quad -r_1\left(b - \tfrac{qx(\mu+d+\gamma)\eta}{\beta(g-\eta)}\right) + (\mu + d + \gamma) + \tfrac{\mu x(\mu+d+\gamma)}{\beta(g-\eta)}, \\ C_0 &= -\tfrac{x(\mu+d+\gamma)\eta}{\beta(g-\eta)}\left((b - \mu) - q\tfrac{x(\mu+d+\gamma)\eta}{\beta(g-\eta)}\right). \end{aligned}$$

Since $C_2 > 0$ and C_0 is negative ($\frac{(b-\mu)}{q} = N_0 > S^* > \frac{x(\mu+d+\gamma)\eta}{\beta(g-\eta)}$), I^* is given by

$$I^* = \frac{-C_1 + \sqrt{C_1^2 - 4C_0C_2}}{2C_2}.$$

1.1.2 The basic reproductive number The basic reproductive number (R_0) has played a crucial role in determining whether an infectious disease can spread through a population. It is defined as the expected number of secondary infections resulting from an introduction of a single infectious individual into a disease-free population (**Anderson and May** (1991)). If $R_0 < 1$, the number of individuals infected by the pathogen declines, and if $R_0 > 1$, the number increases with each generation.

Here, we use a method of the next-generation matrix to calculate R_0. The element (i, j) of the matrix represents the expected number of new infections of type i caused by an (infected) individual of type j (**Diekmann and Heesterbeek** (2000); **Diekmann et al.** (2010)). There are three types of infections in

model (1): I, V_1, and W. Hence, the next generation matrix is

$$K = \begin{bmatrix} 0 & \frac{\theta}{(\mu+\lambda)} & \frac{\beta N^0}{\eta} \\ 0 & 0 & 0 \\ \frac{\alpha M^0}{(\mu+d+\gamma)} & \frac{(1-\sigma)\alpha M}{(\mu+\lambda)} & 0 \end{bmatrix}$$

with $N^0 = (b-\mu)/q$ and $M^0 = (g-\eta)/x$. The basic reproductive number of (1) is then the largest eigenvalue of the matrix K (**Diekmann and Heesterbeek** (2000); **Diekmann et al.** (2010)) and is given by:

$$R_0 = \frac{\beta\alpha M^0 N^0}{(\mu+d+\gamma)\eta}. \tag{3}$$

Note that we use a square of the largest eigenvalue as R_0.

1.1.3 Stability analysis We first establish the stability of the disease-free steady state P^0.

Theorem 1 *The disease-free steady state P^0 of (1) is locally asymptomatically stable in Γ if $R_0 < 1$ and it is unstable if $R_0 > 1$.*

PROOF. This theorem can be proved by the definition of R_0 and theorems in (**Diekmann and Heesterbeek** (2000); **Diekmann et al.** (2010)). We examine this theorem by considering the signs of eigenvalues of the Jacobian matrix of (1) at the disease-free steady state P^0 as follows:

$$J^0 = \begin{bmatrix} b-2qN^0 & -qN^0+r_1b-r_1qN^0 & \begin{array}{c} b-2qN^0-qN^0+r_2b- \\ r_2qN^0+(1-\rho_{12}-\rho_{13}) \end{array} & 0 & 0 & -\beta N^0 \\ 0 & -(\mu+d+\gamma) & 0 & \rho_{13}\lambda & 0 & \beta N^0 \\ 0 & \gamma & -\mu & \rho_{12}\lambda & 0 & 0 \\ 0 & 0 & 0 & -(\mu+\lambda) & 0 & 0 \\ 0 & -\alpha M^0 & 0 & -(1-\delta)\alpha M^0 & g-2xM^0-\eta & g-2xM^0 \\ 0 & \alpha M^0 & 0 & (1-\delta)\alpha M^0 & 0 & -\eta \end{bmatrix}.$$

Eigenvalues of J^0 are given by $\lambda_1 = b-2qN^0-\mu$, $\lambda_2 = -\mu$, $\lambda_3 = g-2xM^0-\eta$, $\lambda_4 = -(\mu+\lambda)$, and

$$\lambda_5 = \frac{-(\mu+d+\gamma+\eta) \pm \sqrt{(\mu+d+\gamma+\eta)^2 + 4\eta(\mu+d+\gamma)(R_0-1)}}{2}.$$

Clearly, all the eigenvalues of J^0 are negative if and only if $R_0 < 1$.

Theorem 2 *If $R_0 < 1$, the disease-free steady state P^0 of (1) is globally asymptomatically stable.*

PROOF. Let us define a function $\mathcal{V}$ as follows:

$$\mathcal{V} = \mu\eta I + \beta\mu N^0 W + \left(\frac{\mu\eta\rho_{13}\lambda + (1-\delta)\beta\alpha\mu M^0 N^0}{(\mu+\lambda)}\right) V_1.$$

Clearly, $\mathcal{V}$ is positive definite as $\mathcal{V}(I, V_1, W) \geq 0$ for all I, V_1, and W and $\mathcal{V}(I, V_1, W) = 0$ if and only if $I = V_1 = W = 0$. Because

$$\begin{aligned} \dot{\mathcal{V}} &= \mu\eta\,(\beta WS - (\mu+d+\gamma)I) + \beta\mu N^0\,(\alpha IU + (1-\delta)\alpha V_1 U - \eta W) - (1-\delta)\beta\alpha\mu M^0 N^0 V_1, \\ &\leq \mu\eta\left(\beta WN^0 - (\mu+d+\gamma)I\right) + \beta\mu N^0\left(\alpha IM^0 + (1-\delta)\alpha V_1 M^0 - \eta W\right) - (1-\delta)\beta\alpha\mu M^0 N^0 V_1, \\ &= \mu(\mu+d+\gamma)\eta(R_0-1)I, \end{aligned}$$

we have $\dot{\mathcal{V}} < 0$ for all $I, V_1, W \neq 0$ and $\dot{\mathcal{V}} = 0$ for $I = V_1 = W = 0$ if $R_0 < 1$. By the LaSalle's invariance principle (**LaSalle** (1976)), $\mathcal{V}$ is a Lyapunov function and consequently the disease-free steady state P^0 of (1) is globally asymptomatically stable if $R_0 < 1$.

Studying stability of P^* in (1) directly is unwieldy and not tractable. Hence, we omit it and will shortly make additional assumptions to study a simpler system in this supplement.

1.1.4 Impacts of vaccinated ruminants Neither the disease-present steady state nor the basic reproductive number gives information of possible consequences from an introduction of vaccinated ruminants with live vaccines into areas in which live vaccines have not been implemented previously. Here, we demonstrate that the introduction of vaccinated ruminants into the areas may lead to a higher number of infectious ruminants.

From (1), we have

$$\dot{I} \geq \rho_{13}\lambda V_1 - (\mu + d + \gamma)I.$$

Hence,

$$d(e^{(\mu+d+\gamma)\tau} I) \geq \rho_{13}\lambda V_1(0) e^{(d+\gamma-\lambda)\tau} d\tau.$$

Integrating both sides on the interval $[0, t]$ gives

$$I \geq \rho_{13}\lambda V_1(0) \frac{(e^{-(\mu+\lambda)t} - e^{-(\mu+d+\gamma)t})}{(d + \gamma - \lambda)}$$

for any $I(0) \geq 0$. Hence, even if $I(0) = 0$, we have $I(t) > 0$ for $t > 0$ if ruminants vaccinated with live vaccines are introduced. Even without the introduction of additional vaccinated or infectious ruminants, having $I(t) > 0$ for some $t > 0$ would lead to an outbreak if $R_0 > 1$. Note that vaccinated ruminants by live vaccines can also transmit RVFV to mosquitoes and consequently increase the number of infectious ruminants.

1.2 WITH VACCINATION ($\rho_{11}, \phi_1 \neq 0$)

When vaccination is present, studying dynamics of (1) directly can be difficult and unwieldy. Let us assume that the total number of ruminants is constant ($N = N^0$) (herdsmen may maintain the size of animal herds), so that $\Lambda_r(S, I, R, V_1) = \mu N^0 + dI$. We also use the theory of asymptomatically autonomous systems to study the qualitatively equivalent system for a mosquito population.

In the absence of RVFV, one has

$$\dot{M} = (g - xM)M - \eta M = \Lambda_m(M) - \eta M.$$

Hence, $\Lambda_m(0) = 0$, $\dot{\Lambda}_m(0) = g > \eta$, and $\ddot{\Lambda}_m(M) = -2x < 0$. The mosquito carrying capacity M^0 satisfies

$$\Lambda_m(M^0) = \eta M^0 \text{ and } \dot{\Lambda}_m(M^0) = -g + 2\eta = (\eta - (g - \eta)) < \eta.$$

The latter condition assures the asymptotic stability of M^0 such that

$$\Lambda_m(M) > \eta M, \text{ for } 0 \leq M \leq M^0.$$

When RVFV is present, $\Lambda_m(M) \leq \Lambda_m(M^0)$. Therefore, under the assumption that $\Lambda_r(S, I, R, V_1) = \mu N^0 + dI$ and by the theory of asymptotically autonomous systems (**Thieme** (1992), **Brauer et al.** (2008)), the system (1) is equivalent to the system in which S is replaced by $N^0 - I - R - V_1$ and M is replaced by its limit as follows:

$$\begin{aligned}
\dot{I} &= \beta W(N^0 - I - R - V_1) + \rho_{13}\lambda V_1 - (\mu + d + \gamma)I, \\
\dot{R} &= \gamma I + \rho_{12}\lambda V_1 - \mu R, \\
\dot{V_1} &= \rho_{11}\phi_1(N^0 - I - R - V_1) - (\mu + \lambda)V_1, \\
\dot{W} &= \alpha I(M^0 - W) + (1 - \delta)\alpha V_1(M^0 - W) - \eta W.
\end{aligned} \tag{4}$$

1.2.1 Steady states As $S = N^0$ and $U = M^0$ when RVFV is absent, there is no disease-free steady state of (4). If a disease-present steady state of (4) in Γ exists (by setting the right hand sides of (4) equal to zero), it is in the following form:

$$(I, R, V_1, W) = P_0^* = (I^*, R^*, V_1^*, W^*)$$

with

$$\begin{aligned}
V_1^* &= h_1 - h_2 I^* \left(h_1 = \frac{\rho_{11}\phi_1 \mu N^0}{\rho_{11}\phi_1(\rho_{12}\lambda + \mu) + \mu(\mu+\lambda)}, h_2 = \frac{\rho_{11}\phi_1(\mu+\gamma)}{\rho_{11}\phi_1(\rho_{12}\lambda+\mu)+\mu(\mu+\lambda)} \right), \\
R^* &= h_3 + h_4 I^* \left(h_3 = \frac{\rho_{12}\lambda h_1}{\mu}, h_4 = \frac{\gamma - \rho_{12}\lambda h_2}{\mu} \right), \\
W^* &= \frac{h_5 + h_6 I^*}{h_7 + h_8 I^*} \ (h_5 = (1-\delta)\alpha M^0 h_1, h_6 = \alpha M^0(1-(1-\delta)h_2), h_7 = (1-\delta)\alpha h_1 + \eta, \\
&\quad h_8 = \alpha(1-(1-\delta)h_2)),
\end{aligned}$$

and I^* satisfying

$$a_2 I^{*2} + a_1 I^* + a_0 = 0,$$

where

$$\begin{aligned}
a_2 &= (\mu + d + \gamma)h_8 + \rho_{13}\lambda h_2 h_8 + \beta h_6(1 + h_4 - h_2), \\
a_1 &= (\mu + d + \gamma)h_7 + \rho_{13}\lambda(h_2 h_7 - h_1 h_8) + \beta[h_5(1 + h_4 - h_2) - h_6(N^0 - h_1 - h_3)], \\
a_0 &= -\rho_{13}\lambda h_1 h_7 - \beta h_5(N^0 - h_1 - h_3).
\end{aligned}$$

It is not obvious that a positive and real root exists for the equation. However, by exploring the value of I^* under certain parameter values numerically, our results suggest that I^* exists and is positive.

1.2.2 Stability analysis Now we prove the stability of the disease-present steady state P_0^*.

Thorem 3 *The disease-present steady state P_0^* of (1) is locally asymptomatically stable in Γ.*

PROOF. We linearize the system (4) at the disease-present steady state. The Jacobian matrix at P_0^* is given by:

$$J^* = \begin{bmatrix} -a_{11} & -a_{12} & -a_{13} & a_{14} \\ a_{21} & -a_{22} & a_{23} & 0 \\ -a_{31} & -a_{32} & -a_{33} & 0 \\ a_{41} & 0 & a_{43} & -a_{44} \end{bmatrix}$$

with $a_{11} = \beta W^* + \mu + d + \gamma,\ a_{12} = \beta W^*,\ a_{13} = (\beta W^* - \rho_{13}\lambda),\ a_{14} = \beta(N^0 - I^* - R^* - V_1^*),\ a_{21} = \gamma,\ a_{22} = \mu,\ a_{23} = \rho_{12}\lambda,\ a_{31} = \rho_{11}\phi_1,\ a_{32} = \rho_{11}\phi_1,\ a_{33} = \rho_{11}\phi_1 + \mu + \lambda,\ a_{41} = \alpha(M^0 - W^*),\ a_{43} = (1-\delta)\alpha(M^0 - W^*),\ a_{44} = \alpha I^* + (1-\delta)\alpha V_1^* + \eta$. The characteristic equation of J^* is

$$z^4 + a_1 z^3 + a_2 z^2 + a_3 z + a_4 = 0$$

with

$$\begin{aligned}
a_1 &= a_{11} + a_{22} + a_{33} + a_{44}, \\
a_2 &= a_{11}a_{22} + a_{11}a_{33} + a_{11}a_{44} + a_{12}a_{21} + a_{22}a_{33} + a_{22}a_{44} + a_{23}a_{32} + a_{33}a_{44} - a_{13}a_{31} - a_{14}a_{41}, \\
a_3 &= a_{11}a_{22}a_{33} + a_{11}a_{22}a_{44} + a_{11}a_{33}a_{44} + a_{11}a_{23}a_{32} + a_{12}a_{21}a_{33} + a_{12}a_{21}a_{44} + a_{14}a_{31}a_{43} \\
&\quad + a_{22}a_{33}a_{44} + a_{23}a_{32}a_{44} - a_{12}a_{23}a_{31} - a_{13}a_{21}a_{32} - a_{13}a_{22}a_{31} - a_{13}a_{31}a_{44} - a_{14}a_{22}a_{41} \\
&\quad - a_{14}a_{33}a_{41}, \\
a_4 &= a_{11}a_{22}a_{33}a_{44} + a_{11}a_{23}a_{32}a_{44} + a_{12}a_{21}a_{33}a_{44} + a_{14}a_{21}a_{32}a_{43} + a_{14}a_{22}a_{31}a_{43} \\
&\quad - a_{12}a_{23}a_{31}a_{44} - a_{13}a_{21}a_{32}a_{44} - a_{13}a_{22}a_{31}a_{44} - a_{14}a_{22}a_{33}a_{41} - a_{14}a_{23}a_{32}a_{41}.
\end{aligned}$$

According to the Routh-Hurwitz criteria, the disease-present steady state is locally asymptomatically stable if and only if

$$a_1 > 0,\ a_3 > 0,\ a_4 > 0,\ \text{ and } a_1a_2a_3 > a_3^2 + a_1^2a_4.$$

If P_0^* exists, $a_{ii} > 0$ for $i = 1, \dots, 4$ so that $a_1 > 0$ and we have $a_{11}a_{23}a_{32} > a_{12}a_{23}a_{31}$, $a_{12}a_{21}a_{33} > a_{13}a_{21}a_{32}$, and $a_{11}a_{22}a_{33} > a_{13}a_{22}a_{31}$. Because $\beta(N^0 - I^* - R^* - V_1^*)\alpha(M^0 - W^*) = \frac{[(\mu+d+\gamma)I^* - \rho_{13}\lambda V_1^*]\eta}{I^* + (1-\delta)V_1^*} \leq (\mu + d + \gamma)\eta$, we obtain $a_{11}a_{33}a_{44} > a_{13}a_{31}a_{44} + a_{14}a_{33}a_{41}$ and $a_{11}a_{22}a_{44} > a_{14}a_{22}a_{41}$. Hence, $a_3 > 0$. For a_4, we have $a_{11}a_{22}a_{33}a_{44} > a_{13}a_{22}a_{31}a_{44} + a_{14}a_{22}a_{33}a_{41}$, $a_{11}a_{23}a_{32}a_{44} > a_{12}a_{23}a_{31}a_{44} + a_{14}a_{23}a_{32}a_{41}$, and $a_{12}a_{21}a_{33}a_{44} > a_{13}a_{21}a_{32}a_{44}$. Consequently, $a_4 > 0$. Analytically showing that $a_1a_2a_3 > a_3^2 + a_1^2a_4$ can be very unwieldy. By using simulations to explore a sign of $a_1a_2a_3 - (a_3^2 + a_1^2a_4)$ under possible values of parameters, it suggests that this quantity is positive. For example, if $1/\mu = 5.7, \tau = 8/365, m = 0.3, 1/\phi = 141/365, \rho_{11} = 0.8, \rho_{12} = 0.9, \rho_{13} = 0.05, 1/\lambda = 21/365, a = 256, p_r = 0.14, p_m = 0.35, 1/\eta = 60/365, k = 1.5$, and $\delta = 0.8$, which is a set of parameters used in several of our simulation results, $a_1a_2a_3 - (a_3^2 + a_1^2a_4) \approx 5.05 \times 10^8$. When parameters are more towards the extinction of RVFV, such as $1/\mu = 10, \tau = 8/365, m = 0.1, 1/\phi = 100/365, \rho_{11} = 1, \rho_{12} = 0.999, \rho_{13} = 0.001, 1/\lambda = 4/365, a = 100, p_r = 0.1, p_m = 0.1, 1/\eta = 10/365, k = 0.5$, and $\delta = 1$, we find $a_1a_2a_3 - (a_3^2 + a_1^2a_4) \approx 9 \times 10^{11}$. When parameters are more towards the RVFV establishment that (for example) $1/\mu = 1, \tau = 30/365, m = 0.9, 1/\phi = 200/365, \rho_{11} = 0, \rho_{12} = 0.999, \rho_{13} = 0.2, 1/\lambda = 30/365, a = 500, p_r = 0.5, p_m = 0.5, 1/\eta = 90/365, k = 10$, and $\delta = 0$, $a_1a_2a_3 - (a_3^2 + a_1^2a_4) \approx 3.6 \times 10^{15}$.

2 ANALYSIS OF THE VACCINATION MODEL WITH KILLED VACCINES

Let us consider the following model:

$$\begin{aligned}
\dot{S} &= \Lambda_r(S, I, R, V_2) + (1-\rho_{22})\nu V_2 - \beta W S - \rho_{21}\phi_2 S - \mu S,\\
\dot{I} &= \beta W S + (1-\sigma)\beta W V_2 - (\mu + d + \gamma)I,\\
\dot{R} &= \gamma I + \rho_{22}\nu V_2 - \mu R,\\
\dot{V_2} &= \rho_{21}\phi_2 S - (1-\sigma)\beta W V_2 - (\mu + \nu)V_2,\\
\dot{U} &= \Lambda_m(M) - \alpha I U - \eta U,\\
\dot{W} &= \alpha I U - \eta W
\end{aligned} \tag{5}$$

with $\Lambda_r(S, I, R, V_2) = (b - qN)(S + R + V_2) + r_1(b - qN)I$, $\Lambda_m(M) = (g - xM)M$, $q = (b - \mu)/N^0$ (where N^0 is the carrying capacity of a ruminant population), $x = (g - \eta)/M^0$ (where M^0 is the carrying capacity of a mosquito population), $b > \mu$, and $g > \eta$. We define the closed and positively invariant set:

$$\Gamma^k = \left\{ (S, I, R, V_2, U, W) \in \mathcal{R}_+^6 \middle| \begin{array}{l} S + I + R + V_2 \leq \frac{(b-\mu)}{q}, U + W \leq \frac{(g-\eta)}{x}, \\ S \geq 0, I \geq 0, R \geq 0, V_2 \geq 0, U \geq 0, W \geq 0 \end{array} \right\}.$$

2.1 STEADY STATES

There are five steady states of the system (5):

1. $P^0 = (0, 0, 0, 0, 0, 0)$ is a disease-free steady state with extinction of both ruminant and mosquito populations;
2. $P^1 = (0, 0, 0, 0, (g - \eta)/x, 0)$ is a disease-free steady state with extinction of a ruminant population;

3. $P^2 = (S^2, 0, R^2, V_2^2, 0, 0)$ is a disease-free steady state with extinction of a mosquito population

$$\begin{aligned} S^2 &= \frac{(b-\mu)\mu}{q[\rho_{21}\rho_{22}\phi_2\nu+\mu\rho_{21}\phi_2+\mu(\mu+\nu)]}, \\ R^2 &= \frac{(b-\mu)\rho_{21}\rho_{22}\nu\phi_2}{q[\rho_{21}\rho_{22}\phi_2\nu+\mu\rho_{21}\phi_2+\mu(\mu+\nu)]}, \\ V_2^2 &= \frac{(b-\mu)\mu\rho_{21}\phi_2}{q[\rho_{21}\rho_{22}\phi_2\nu+\mu\rho_{21}\phi_2+\mu(\mu+\nu)]}; \end{aligned}$$

4. $P^3 = (S^3, 0, R^3, V_2^3, (g-\eta)/x, 0)$ is a disease-free steady state with vaccine administration in a ruminant population ($S^3 = S^2, R^3 = R^2$, and $V_2^3 = V_2^2$);
5. $P^* = (S^*, I^*, R^*, V_2^*, U^*, W^*)$ is a disease-present steady state

$$\begin{aligned} S^* &= \left[\frac{(1-\sigma)(g-\eta)\beta\alpha I^*}{\rho_{21}\phi_2 x(\eta+\alpha I^*)} + \frac{(\mu+\nu)}{\rho_{21}\phi_2}\right]\left[\frac{\rho_{21}\phi_2 x^2(\mu+d+\gamma)(\eta+\alpha I^*)^2}{(g-\eta)\beta\alpha[(1-\sigma)(g-\eta)\beta\alpha I^*+(\mu+\nu)x(\eta+\alpha I^*)+(1-\sigma)\rho_{21}\phi_2 x(\eta+\alpha I^*)]}\right], \\ R^* &= \frac{\gamma I^*(g-\eta)\beta\alpha[(1-\sigma)(g-\eta)\beta\alpha I^*+(\mu+\nu)x(\eta+\alpha I^*)+(1-\sigma)\rho_{21}\phi_2 x(\eta+\alpha I^*)]+\rho_{21}\rho_{22}\nu\phi_2 x^2(\mu+d+\gamma)(\eta+\alpha I^*)^2}{\mu(g-\eta)\beta\alpha[(1-\sigma)(g-\eta)\beta\alpha I^*+(\mu+\nu)x(\eta+\alpha I^*)+(1-\sigma)\rho_{21}\phi_2 x(\eta+\alpha I^*)]}, \\ V_2^* &= \frac{\rho_{21}\phi_2 x^2(\mu+d+\gamma)(\eta+\alpha I^*)^2}{(g-\eta)\beta\alpha[(1-\sigma)(g-\eta)\beta\alpha I^*+(\mu+\nu)x(\eta+\alpha I^*)+(1-\sigma)\rho_{21}\phi_2 x(\eta+\alpha I^*)]}, \\ U^* &= \frac{(g-\eta)\eta}{x(\eta+\alpha I^*)}, \\ W^* &= \frac{(g-\eta)\alpha I^*}{x(\eta+\alpha I^*)}, \end{aligned}$$

where I^* satisfies the following equation:

$$\begin{aligned} &(b - q(S^* + I^* + R^* + V_2^*)) + (S^* + R^* + V_2^*) + r_1(b - q(S^* + I^* + R^* + V_2^*))I^* \\ &\quad +(1-\rho_{22})\nu V_2^* - \beta W^* S^* - \rho_{21}\phi_2 S^* - \mu S^* = 0. \end{aligned}$$

Theorem 4 *The disease-free steady state P^0 of (5) is unstable in Γ^k.*

PROOF. We linearize system (5) at P^0. The Jacobian matrix at P^0 is given by

$$J^0 = \begin{bmatrix} b-\rho_{21}\phi_2-\mu & r_1 b & b & b+(1-\rho_{22})\nu & 0 & 0 \\ 0 & -(\mu+d+\gamma) & 0 & 0 & 0 & 0 \\ 0 & \gamma & -\mu & \rho_{22}\nu & 0 & 0 \\ \rho_{21}\phi_2 & 0 & 0 & -(\mu+\nu) & 0 & 0 \\ 0 & 0 & 0 & 0 & g-\eta & g \\ 0 & 0 & 0 & 0 & 0 & -\eta \end{bmatrix}.$$

Eigenvalues of J^0 are $g-\eta$, $-\eta$, $-(\mu+d+\gamma)$, and those of

$$\hat{J} = \begin{bmatrix} b-\rho_{21}\phi_2-\mu & b & b+(1-\rho_{22})\nu \\ 0 & -\mu & \rho_{22}\nu \\ \rho_{21}\phi_2 & 0 & -(\mu+\nu) \end{bmatrix}.$$

Because $g > \eta$, the eigenvalue $g-\eta$ of (5) is always positive and consequently P^0 is unstable.

As studying stability of other steady states in (5) can be unwieldy and not tractable, we omit it and investigate the basic reproductive number of (5) and a simpler system under some additional assumptions.

2.2 THE BASIC REPRODUCTIVE NUMBER

By considering the next-generation matrix of (5), there are two types of infections: I and W. The next generation matrix is given by

$$K = \begin{bmatrix} 0 & \frac{\beta(N^0-R^3-V_2^3)+(1-\sigma)\beta V_2^3}{\eta} \\ \frac{\alpha M^0}{(\mu+d+\gamma)} & 0 \end{bmatrix}$$

with $N^0 = (b-\mu)/q$ and $M^0 = (g-\eta)/x$. The basic reproductive number of (5) is the largest eigenvalue of the K matrix and is described by:

$$R_0^k = \frac{\beta\alpha M^0 N^0 \mu(\mu+\nu) + (1-\sigma)\beta\alpha M^0 N^0 \mu\rho_{21}\phi_2}{\eta(\mu+d+\gamma)[\rho_{21}\rho_{22}\phi_2\nu + \mu\rho_{21}\phi_2 + \mu(\mu+\nu)]}. \tag{6}$$

Note that we use a square of the largest eigenvalue as R_0^k. From the definition of R_0^k and theorems **Diekmann and Heesterbeek** (2000) and **Diekmann et al.** (2010), the disease-free steady state P^2 of (5) is locally asymptomatically stable in Γ^k if $R_0 < 1$ and unstable if $R_0 > 1$.

2.3 CONSTANT NUMBERS OF RUMINANTS

Let us assume that the total number of ruminants is constant in (5) ($N = N^0$), that $\Lambda_r(S, I, R, V_2) = \mu N^0 + dI$ and $S = N^0 - I - R - V_2$. We use the theory of asymptomatically autonomous systems for a mosquito population so that U can be replaced by $M^0 - W$ (see Section 1 for further details).

$$\begin{aligned} \dot{I} &= \beta W(N^0 - I - R - V_2) + (1-\sigma)\beta W V_2 - (\mu+d+\gamma)I, \\ \dot{R} &= \gamma I + \rho_{22}\nu V_2 - \mu R, \\ \dot{V_2} &= \rho_{21}\phi_2(N^0 - I - R - V_2) - (1-\sigma)\beta W V_2 - (\mu+\nu)V_2, \\ \dot{W} &= \alpha I(M^0 - W) - \eta W. \end{aligned} \tag{7}$$

2.3.1 Steady states There are three steady states of (7):

1. $P_0^0 = (I, R, V_2, W) = (0, 0, 0, 0)$ is a disease-free steady state with no administration of vaccination;
2. $P_0^1 = (I, R, V_2, W) = (0, R^1, V_2^1, 0) = \left(0, \frac{\rho_{21}\rho_{22}\phi_2\nu N^0}{\rho_{21}\rho_{22}\phi_2\nu+\mu\rho_{21}\phi_2+\mu(\mu+\nu)}, \frac{\mu\rho_{21}\phi_2 N^0}{\rho_{21}\rho_{22}\phi_2\nu+\mu\rho_{21}\phi_2+\mu(\mu+\nu)}, 0\right)$ is a disease-free steady state with administration of vaccination;
3. $P_0^1 = (I, R, V_2, W) = (I^*, R^*, V_2^*, W^*)$ is a disease-present steady state with

$$\begin{aligned} R^* &= \frac{\gamma I^*}{\mu} + \frac{\rho_{21}\rho_{22}\phi_2\nu[\mu(N^0-I^*)-\gamma I^*][\alpha I^*+\eta]}{\mu[(\alpha I^*+\eta)(\rho_{21}\rho_{22}\phi_2\nu+\rho_{21}\phi_2\mu+\mu(\mu+\nu))+\mu(1-\sigma)\beta\alpha I^* M^0]}, \\ V_2^* &= \frac{\rho_{21}\phi_2[\mu(N^0-I^*)-\gamma I^*][\alpha I^*+\eta]}{(\alpha I^*+\eta)(\rho_{21}\rho_{22}\phi_2\nu+\rho_{21}\phi_2\mu+\mu(\mu+\nu))+\mu(1-\sigma)\beta\alpha I^* M^0}, \\ W^* &= \frac{\alpha I^* M^0}{\alpha I^*+\eta}, \end{aligned}$$

where I^* satisfies the following equation:

$$\beta W^*(N^0 - I^* - R^* - V_2^*) + (1-\sigma)\beta W^* V_2^* - (\mu+d+\gamma)I^* = 0,$$

which leads to a polynomial of degree 3 in I^*.

2.3.2 Stability Because P_0^0 can be obtained from P_0^1 by setting $\phi_2 = 0$ or $\rho_{21} = 0$, we only study the stability of P_0^1 in (7).

Theorem 5 *The disease-free steady state P_0^1 of (5) is locally stable in Γ^k if and only if $R_0^k < 1$.*

PROOF. This theorem can be proved by the definition of R_0 and theorems in **Diekmann and Heesterbeek** (2000) and **Diekmann et al.** (2010). We examine this theorem by using the Routh-Hurwitz criterion. The Jacobian matrix of (7) at P_0^1 is as follows:

$$J^1 = \begin{bmatrix} -(\mu+d+\gamma) & 0 & 0 & \beta(N^0 - R^1 - V_2^1) + (1-\sigma)\beta V_2^1 \\ \gamma & -\mu & \rho_{22}\nu & 0 \\ -\rho_{21}\phi_2 & -\rho_{21}\phi_2 & -(\rho_{21}\phi_2 + \mu + \nu) & -(1-\sigma)V_2^1 \\ \alpha M^0 & 0 & 0 & -\eta \end{bmatrix}.$$

The characteristic equation of J^1 is given by:

$$\lambda^4 + a_1\lambda^3 + a_2\lambda^2 + a_3\lambda + a_4 = 0$$

with

$$\begin{aligned} a_1 &= (\mu+d+\gamma+\eta) + (2\mu+\nu+\rho_{21}\phi_2), \\ a_2 &= \eta(\mu+d+\gamma) + (\mu+d+\gamma+\eta)(2\mu+\nu+\rho_{21}\phi_2) + (\rho_{21}\rho_{22}\phi_2\nu + \mu\rho_{21}\phi_2 + \mu(\mu+\nu)) \\ &\quad -\beta\alpha M^0(N^0 - R^1 - V_2^1 + (1-\sigma)V_2^1), \\ a_3 &= \eta(\mu+d+\gamma)(2\mu+\nu+\rho_{21}\phi_2) + (\mu+d+\gamma+\eta)(\rho_{21}\rho_{22}\phi_2\nu + \mu\rho_{21}\phi_2 + \mu(\mu+\nu)) \\ &\quad -(2\mu+\nu+\rho_{21}\phi_2)\beta\alpha M^0(N^0 - R^1 - V_2^1 + (1-\sigma)V_2^1), \\ a_4 &= \eta(\mu+d+\gamma)(\rho_{21}\rho_{22}\phi_2\nu + \mu\rho_{21}\phi_2 + \mu(\mu+\nu)) \\ &\quad -(\rho_{21}\rho_{22}\phi_2\nu + \mu\rho_{21}\phi_2 + \mu(\mu+\nu))\beta\alpha M^0(N^0 - R^1 - V_2^1 + (1-\sigma)V_2^1). \end{aligned}$$

By the Routh-Hurwitz criteria, P_0^1 is locally asymptomatically stable if and only if

$$a_1 > 0,\ a_3 > 0,\ a_4 > 0, \text{ and } a_1a_2a_3 > a_3^2 + a_1^2a_4.$$

Under the assumption that all parameters have positive values, it is clear that $a_1 > 0$. Since

$$N^0 - R^1 - V_2^1 + (1-\sigma)V_2^1 = \frac{(\mu(\mu+\nu) + (1-\sigma)\mu\rho_{21}\phi_2)N^0}{(\rho_{21}\rho_{22}\phi_2\nu + \mu\rho_{21}\phi_2 + \mu(\mu+\nu))},$$

we can rewrite a_2 and a_4 in terms of R_0^k and other parameters as follows

$$\begin{aligned} a_3 &= \eta(\mu+d+\gamma)(2\mu+\nu+\rho_{21}\phi_2) + (\mu+d+\gamma+\eta)(\rho_{21}\rho_{22}\phi_2\nu + \mu\rho_{21}\phi_2 + \mu(\mu+\nu)) \\ &\quad -\eta(\mu+d+\gamma)(2\mu+\nu+\rho_{21}\phi_2)R_0^k, \end{aligned}$$

and

$$a_4 = \eta(\mu+d+\gamma)(\rho_{21}\rho_{22}\phi_2\nu + \mu\rho_{21}\phi_2 + \mu(\mu+\nu))(1 - R_0^k).$$

Consequently, if $R_0^k < 1$, $a_3 > 0$ and $a_4 > 0$. Moreover, if $R_0^k > 1$, $a_4 < 0$ and P_0^1 is unstable. After canceling some terms out, we obtain

$$\begin{aligned} a_1a_2a_3 - (a_3^2 + a_1^2a_4) \quad = \quad & \eta(\mu+d+\gamma)(\mu+d+\gamma+\eta)(2\mu+\nu+\rho_{21}\phi_2)(1-R_0^k)^2 \\ & +\eta(\mu+d+\gamma)(\mu+d+\gamma+\eta)^2(2\mu+\nu+\rho_{21}\phi_2)^2(1-R_0^k) \\ & +\eta(\mu+d+\gamma)(\mu+d+\gamma+\eta)(2\mu+\nu+\rho_{21}\phi_2)^3(1-R_0^k) \\ & +(\mu+d+\gamma+\eta)^2(2\mu+\nu+\rho_{21}\phi_2)^2(\rho_{21}\rho_{22}\phi_2\nu + \mu\rho_{21}\phi_2 + \mu(\mu+\nu)) \\ & +(\mu+d+\gamma+\eta)(2\mu+\nu+\rho_{21}\phi_2)(\rho_{21}\rho_{22}\phi_2\nu + \mu\rho_{21}\phi_2 + \mu(\mu+\nu))^2 \\ & +2\eta(\mu+d+\gamma)(\mu+d+\gamma+\eta)(2\mu+\nu+\rho_{21}\phi_2)R_0^k \\ & +(2\mu+\nu+\rho_{21}\phi_2)(\rho_{21}\rho_{22}\phi_2\nu + \mu\rho_{21}\phi_2 + \mu(\mu+\nu)) \\ & \quad \times[(\mu+d+\gamma+\eta)^3 - 2\eta(\mu+d+\gamma)(\mu+d+\gamma+\eta)], \end{aligned}$$

which is always positive if $R_0^k < 1$. By Routh-Hurwitz criterion, P_0^1 of (5) is stable if and only if $R_0^k < 1$.

Proving that the disease-present steady state P^* of model (5) is locally stable in Γ^k if and only if $R_0^k > 1$ can be unwieldy but can be done by using Routh-Hurwitz criterion and numerical studies of some criterion (see Section 1 for live vaccines as an example). However, we omit the proof here.

3 ADDITIONAL RESULTS

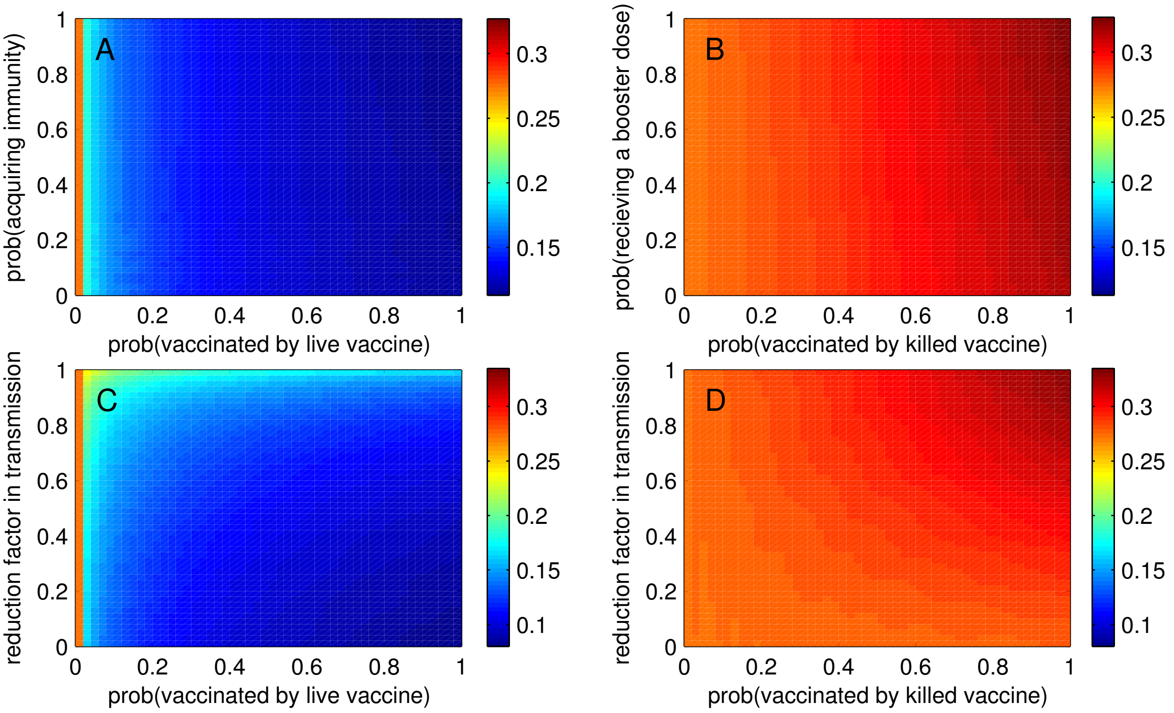

Figure 1. Time from the beginning of an outbreak to a peak when (A) live vaccines are administered and the probability that ruminants are vaccinated (ρ_{11}) and the probability that they acquire immunity (ρ_{12}) vary; (B) killed vaccines are used and the probability that ruminants are vaccinated (ρ_{21}) and the probability that they receive repeated doses (ρ_{22}) vary; (C) live vaccines are used and ρ_{11} and the reduction factor of transmission in vaccinated ruminants (δ) vary; and (D) killed vaccines are used and ρ_{21} and the reduction factor of transmission in vaccinated ruminants (σ) vary.

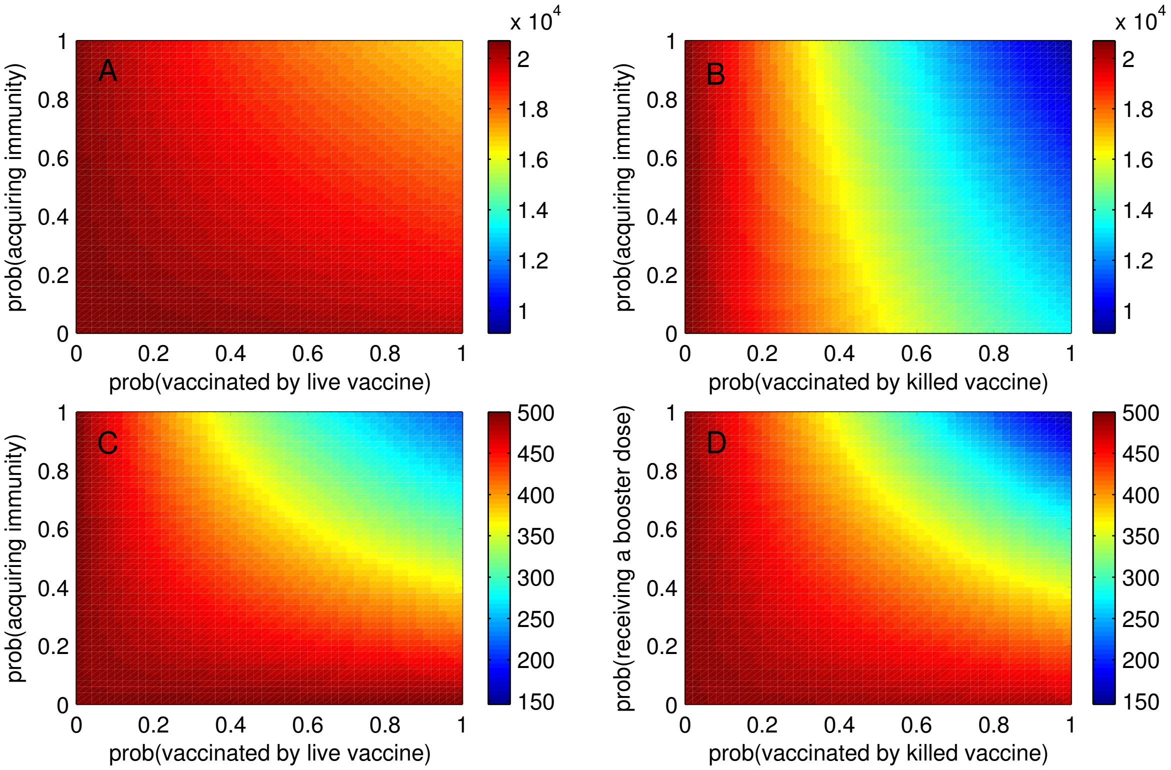

Figure 2. Epidemic size and endemic numbers when none of ruminants are vaccinated before an outbreak: (A) the epidemic size of an outbreak for live vaccines when the probability that ruminants are vaccinated (ρ_{11}) and the probability that they acquire immunity (ρ_{12}) vary; (B) the epidemic size of an outbreak for killed vaccines when the probability that ruminants are vaccinated (ρ_{21}) and the probability that they receive repeated doses (ρ_{22}) vary; (C) the endemic number for live vaccines when ρ_{11} and ρ_{12} vary; and (D) the endemic number for killed vaccines according to the changes of ρ_{21} and ρ_{22}.

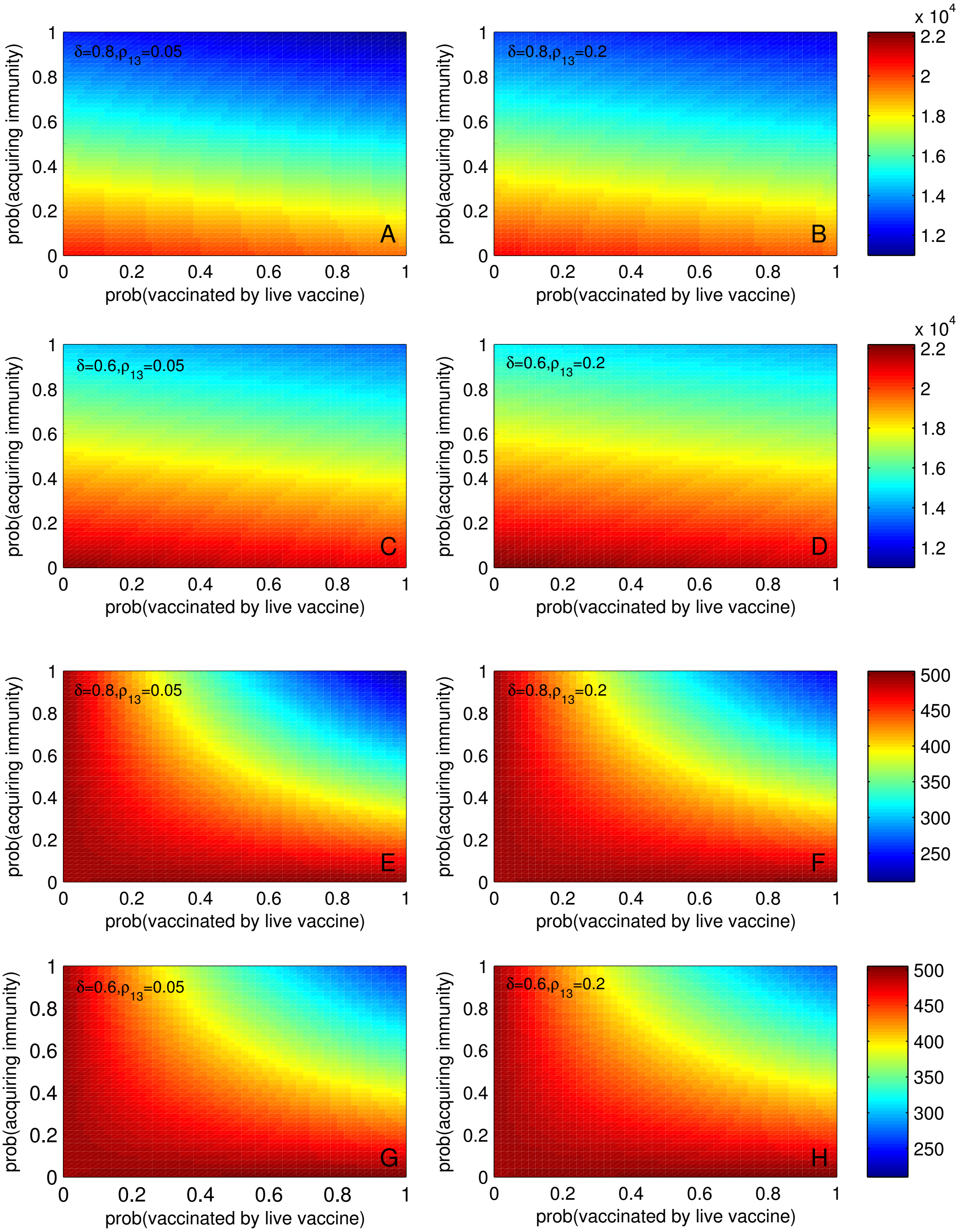

Figure 3. Epidemic size and endemic numbers with different vaccination strategies and vaccine efficacy of live vaccines. (A)-(D) show the epidemic sizes according to the changes of the probability that ruminants are vaccinated by live vaccines (ρ_{11}), the probability that they acquire immunity (ρ_{12}), and vaccine efficacy where (A) the reduction factor of transmission from ruminants to mosquitoes (δ) is 0.8 and the probability of reversion to virulence (ρ_{13}) is 0.05; (B) $\delta = 0.8$ and $\rho_{13} = 0.2$; (C) $\delta = 0.6$ and $\rho_{13} = 0.05$; (D) $\delta = 0.6$ and $\rho_{13} = 0.2$. (E)-(H) show the endemic number according to the changes of ρ_{11} and ρ_{12}, and vaccine efficacy where (E) $\delta = 0.8$ and $\rho_{13} = 0.05$; (F) $\delta = 0.8$ and $\rho_{13} = 0.2$; (G) $\delta = 0.6$ and $\rho_{13} = 0.05$; and (H) $\delta = 0.6$ and $\rho_{13} = 0.2$.

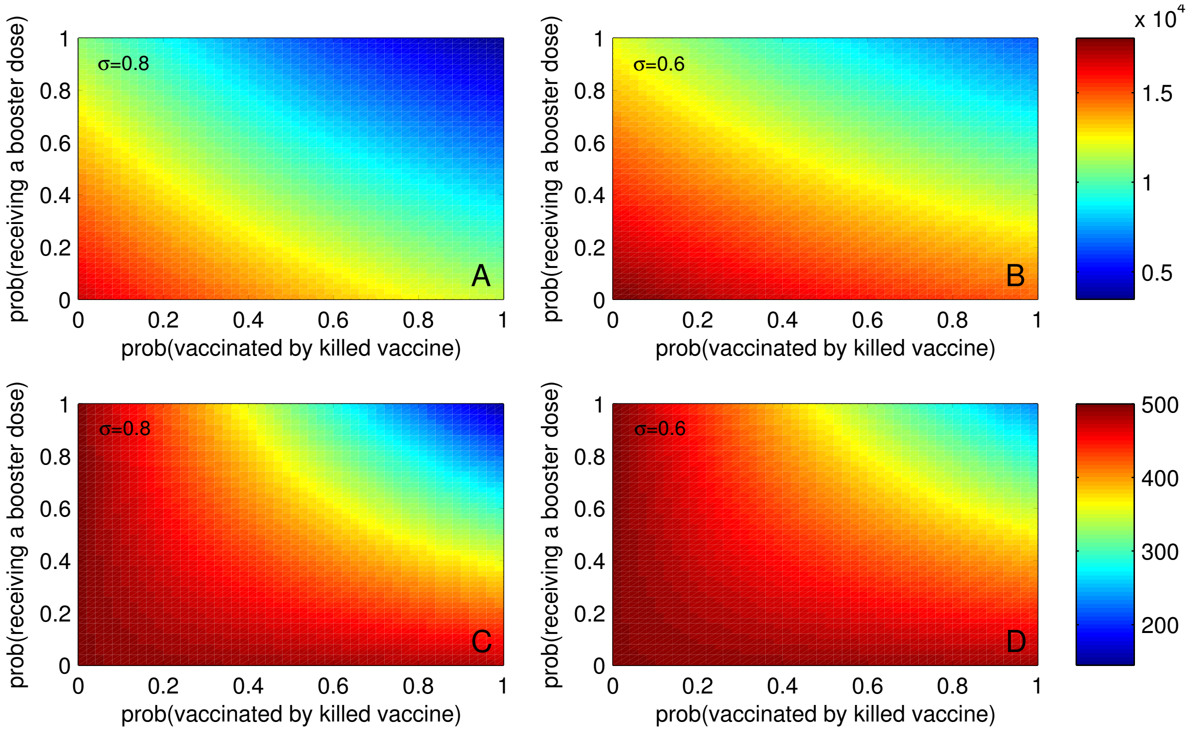

Figure 4. Epidemic size and endemic numbers with different vaccination strategies and vaccine efficacy of killed vaccines. (A)-(B) show the epidemic sizes according to the changes of the probability that ruminants are vaccinated by killed vaccines (ρ_{21}) and the probability that they receive repeated doses (ρ_{22}) when the reduction factor of transmission in ruminants vaccinated by killed vaccines (σ) is 0.8 and 0.6, respectively. (C)-(D) show the endemic numbers according to the changes of ρ_{21} and ρ_{22} with $\sigma = 0.8$ and 0.6, respectively.

REFERENCES

Anderson, R. M. and May, R. M. (1991), Infectious diseases of humans (Oxford University Press, Oxford)

Brauer, F., van den Driessche, P., and Wu, J., eds. (2008), Mathematical Epidemiology (Springer, Berlin, Heidelberg)

Diekmann, O. and Heesterbeek, J. A. P. (2000), Mathematical Epidemiology of Infectious Diseases, Model Building, Analysis and Interpretation (Wiley, Chichester)

Diekmann, O., Heesterbeek, J. A. P., and Roberts, M. G. (2010), The construction of next-generation matrices for compartmental epidemic models, *J. R. Soc. Interface*, 7, 873–885

LaSalle, J. P. (1976), The Stability of Dynamical Systems (SIAM, Philadephia, PA)

Thieme, H. R. (1992), Convergence results and a poincaré-bendixson trichotomy for asymptotically autonomous differential equations, *J. Math. Biol.*, 30, 7, 755–763
